# Expression regulation and functional analysis of RGS2 and RGS4 in adipogenic and osteogenic differentiation of human mesenchymal stem cells

**DOI:** 10.1186/s40659-017-0148-1

**Published:** 2017-12-26

**Authors:** Alma Madrigal, Lun Tan, Yuanxiang Zhao

**Affiliations:** 1 Biological Sciences Department, California State Polytechnic University at Pomona, 3801 W. Temple Ave., Pomona, CA 91768 USA; 2 Present Address: Center for Biomedicine and Genetics, Beckman Research Institute of City of Hope, 1500 E. Duarte Rd., Duarte, CA 91010 USA

**Keywords:** RGS2, RGS4, Human mesenchymal stem cells (hMSCs), Adipogenesis, Osteogenesis

## Abstract

**Background:**

Understanding the molecular basis underlying the formation of bone-forming osteocytes and lipid-storing adipocytes will help provide insights into the cause of disorders originating in stem/progenitor cells and develop therapeutic treatments for bone- or adipose-related diseases. In this study, the role of RGS2 and RGS4, two members of the regulators of G protein signaling (RGS) family, was investigated during adipogenenic and osteogenenic differentiation of human mesenchymal stem cells (hMSCs).

**Results:**

Expression of RGS2 and RGS4 were found to be inversely regulated during adipogenesis induced by dexamethasone (DEX) and 3-isobutyl-methylxanthine, regardless if insulin was present, with RGS2 up-regulated and RGS4 down-regulated in response to adipogenic induction. RGS2 expression was also up-regulated during osteogenesis at a level similar to that induced by treatment of DEX alone, a shared component of adipogenic and osteogenic differentiation inducing media, but significantly lower than the level induced by adipogenic inducing media. RGS4 expression was down-regulated during the first 48 h of osteogenesis but up-regulated afterwards, in both cases at levels similar to that induced by DEX alone. Expression knock-down using small interfering *RNA* against *RGS2* resulted in decreased differentiation efficiency during both adipogenesis and osteogenesis. On the other hand, expression knock-down of *RGS4* also resulted in decreased adipogenic differentiation but increased osteogenic differentiation.

**Conclusions:**

RGS2 and RGS4 are differentially regulated during adipogenic and osteogenic differentiation of hMSCs. In addition, both RGS2 and RGS4 play positive roles during adipogenesis but opposing roles during osteogenesis, with RGS2 as a positive regulator and RGS4 as a negative regulator. These results imply that members of RGS proteins may play multifaceted roles during human adipogenesis and osteogenesis to balance or counterbalance each other’s function during those processes.

**Electronic supplementary material:**

The online version of this article (10.1186/s40659-017-0148-1) contains supplementary material, which is available to authorized users.

## Background

Advancement in understanding adipose and bone tissue biology will help develop new strategies for the prevention and intervention of adipose- and bone-related diseases, including obesity and osteoporosis. Adipogenesis and osteogenesis are processes in which uncommitted stem cells differentiate into mature adipocytes or osteocytes, respectively. Over the past two decades or so, many individual adipogenic regulators have been independently uncovered, which include specific signaling pathways (TGFβ/BMP, Wnt, Hedgehogs, MAPK and JAK-STAT3 signaling etc.), growth factors or cytokines (FGF1/2, RB, ZFP423 and SOX9 etc.), transcription factors (C/EBPs, PPARγ, KLF4, FOXC2 and GATA2/3 etc.), GTPase proteins and its regulators (RHO and ROCK etc.), epigenetic regulators and microRNAs etc. [1–3]. Most significantly, C/EBPα (CCAAT/enhancer binding protein alpha) and PPARγ (peroxisome proliferator-activated receptor gamma) were identified as two key transcriptional factors, which when over-expressed could dictate adipogenic cell fate in both murine preadipocyte cell line 3T3L1 and hMSCs [4–8]. Similarly, many signaling pathways including TGFβ/BMP signaling, Wnt signaling, HH signaling, Notch signaling, PI3K signaling, and ERK1/2 and p38 MAPK mediated signaling, as well as growth factors (FGFs), hormones (Estrogen and Parathyroid hormone), transcription factors (Runx2 and Osterix), bone matrix proteins (ALP, BSP, OCN, OPN, COL 1) and microRNAs etc. have been implicated in osteogenic regulation [9–12]. Among those, Runx 2 and Osterix were identified as master regulators of osteoblast commitment, proliferation and maturation, as knockout mice deficient in either Runx2 or Osterix failed to form bone due to lack of osteoblasts [13, 14]. In addition, overexpression of Runx2 helps stimulate transdifferentiation of 3T3L1 preadipocytes into bone-forming osteoblasts in vitro [15]. Runx2 regulates the expression of osteogenic markers ALP, BSP, OCN, OPN and COL 1, as well as Osterix, though Osterix can be induced by signaling pathways independent of Runx2 [16].

While the adipogenic and osteogenic lineage commitment clearly involve distinct master transcriptional regulators and downstream genetic cascades, in many cases, they are also regulated by the same signaling pathways (Ex. Wnt, IGF and HH signaling) and genes [17, 18]. For examples, many of the *siRNA* hits identified through a high throughput screen were found to promote osteogenic differentiation but inhibited adipogenesis, and cAMP was identified to play opposing roles in osteogeneis vs. adipogenesis [17]. In addition, there appears to be an inverse relationship between adipogenesis and osteogenesis, with one process inhibiting the other [19]. Some medical drugs such as Rosiglitazone have been found to increase adiposity at the expense of bone formation [20–23]. Aging has also been shown to increase bone marrow adiposity but decrease bone mass and strength, which appear to be mediated through regulators of both adipogenesis and osteogenesis [24–28]. These findings indicate that studying the role of potential regulators involved in both adipogeneis and osteogenesis is important in order to better understand the relationship between adipose and bone biology and the etiology of their disease states.

Human mesenchymal stem cells (hMSCs) are a type of adult stem cell that exist in multiple tissues in the body, including adipose tissue, bone marrow and peripheral blood, and play important roles in maintaining normal tissue homeostasis. They can be isolated, expanded and differentiated in vitro into a number of specialized cell types including adipocytes and osteocytes, which makes them an excellent in vitro cell model for studying human adipogenesis and osteogenesis [29]. Using a simple cocktail of adipogenic inducing media (AIM) containing dexamethasone (DEX), 3-isobutyl-1-methylxanthine (IBMX) and insulin, hMSCs can be induced to differentiate into mature adipocytes [29, 30]. Similarly, using a cocktail of osteogenic inducing media (OIM) containing DEX, ascorbic acid-2-phosphate and beta-glycerophosphate, hMSCs can undergo osteogenesis and become mature osteocytes [17, 29]. Due to their ability to differentiate into a variety of mature cell types, low allogeneic immune response and low tumorigenicity in graft recipients, hMSCs have been of great interests to researchers exploring cell-based therapies as well and is the most prevalent cell type used in ongoing stem-cell based clinical trials [31]. In addition to advancing our basic understanding of adipose and bone tissue biology, the potential therapeutic application of hMSCs in adipose and bone tissue engineering makes it even more relevant to use these cells for studying human adipogenesis and osteogenesis [32–35].

Using adipose tissue derived hMSCs as an in vitro model for adipogenic differentiation, we identified through microarray analysis two members of the regulator of G protein signaling (RGS) family, RGS2 and RGS4, which were differentially regulated upon adipogenic induction (unpublished data). Both RGS2 and RGS4 belong to the B/R4 subfamily of RGS proteins family characterized by a conserved 120 aas RGS domain flanked by short amino and carboxyl termini [36]. They are intracellular proteins primarily recognized for their GTPase activating protein (GAPs) activity, which inhibits G-protein coupled receptor (GPCR) signaling by deactivating the Gα subunits of heterotrimeric G proteins through stimulating Gα-bound GTP hydrolysis [37]. RGS2 possesses intrinsic GAP activity that is selective for G_q_-class Gα subunits, whereas RGS4 has intrinsic GAP activity for both G_q_ and G_i/o_-class Gα subunits [38, 39]. As GPCRs comprise the largest cell surface receptors in mammalian cells, GPCR mediated signaling regulates a wide array of cellular processes including proliferation, differentiation, cell death and numerous physiological functions. Unsurprisingly, RGS proteins are expressed in essentially all cell types, tissues and organ systems and have been implicated in various physiology and disease as well, including hemapopoiesis, synaptic signaling plasticity in the brain/anxiety disorder, smooth muscle contraction and relaxation/hypertension, kidney function, cancer migration and invasion [40–45]. Aside from G-protein dependent activity, RGS proteins are also involved in G-protein independent signaling [46].

Despite knowledge in a wide array of biological events involving RGS proteins, our current understanding of the role of RGS proteins during human adipogenesis and osteogenesis is very limited. No study thus far has directly examined the role of RGS in osteogenesis, though past studies have revealed an important role of RGS proteins during bone remodeling by modulating osteoclastogenesis [47, 48]. RGS2 was also found to be expressed in rat metaphyseal and diaphyseal bone and cultured mouse osteoblasts, implicating potential function in bone development [47]. In addition, several GPCRs involved in osteogenesis including parathyroid hormone 1 receptor (PTH1R), frizzled (Fz) and calcium sensing receptor (CsR) are expressed in osteoblasts and regulated by RGS proteins [49]. On the other hand, a more direct relationship between RGS proteins and adipose physiology have been established through knock-out mice models and in vitro studies in murine cell lines. Loss of RGS5 in mice resulted in exacerbated obesity, hepatic steatosis, inflammation, and insulin resistance while loss of RGS2 in mice leads to lower weights, reduced fat deposits, decreased serum lipids, and lower leptin levels [50, 51]. Preadipocytes isolated from *RGS2*−*/*− mice showed lower expression levels of adipogenic markers including PPARYγ, CEBPα, and leptin. In another study using NIH-3T3 mouse preadipocyte cells, RGS2 overexpression promoted adipogensis in the presence of a ligand for PPAR γ [52]. *RGS4* knockout mice also showed a significantly lower body weight compared to wild type mice [53], though in a separate study, these mice showed no significant effect on body weight but had increased circulating free fatty acid, indicating a role in lipolysis [54]. The role of RGS2 or RGS4 in human adipogenesis however remains unknown.

In this study, we characterized the temporal expression patterns of both RGS2 and RGS4 genes during adipogenic and osteogenic differentiation of hMSCs, as well as their function during both processes. Our results demonstrated that RGS2 and RGS4 are differentially regulated during adipogenic and osteogenic differentiation of hMSCs, with both playing positive roles during adipogenesis but opposite roles during osteogenesis.

## Results

### Characterization of adipose-derived hMSCs by clonogenicity and molecular marker expression

The adipose-derived hMSCs used in this study were obtained from a commercial source (see “Methods”). Previously we have shown that these cells were able to differentiate into adipocytes and osteocytes in response to appropriate external stimuli [55]. To gain a better understanding of these cells, both clonogenicity of these cells as well as the expression of three known hMSCs markers, CD73, CD90 and CD105, were examined at passage 4 (P4), the same passage cells used in all subsequent experiments.

Of 3 independent repeats, the average clonogenicity (number of cells per clone is > 50) was determined to be 8% (± 0.67%) (Additional file 1: Figure S1).

To determine the expression of the three marker genes, immunostaining was carried out first. Both CD73 and CD105 were shown to be expressed almost ubiquitously, especially with CD105 whose expression in each individual cell can be easily discerned (Additional file 2: Figure S2, top 2 rows). Immunostaining with antibodies against CD90 from two different sources appeared to be challenging despite several attempts with various conditions, which had also been predicted due to indicated formalin sensitivity of epitope recognized by the antibody. Flow cytometry was subsequently used to analyze the expression of CD90. As control, cells were also co-stained with antibody against CD73. About 96 and 95.72% of cells independently express CD73 and CD90 respectively, and about 95% of cells co-express both (Additional file 2: Figure S2, bottom 2 rows).

In conclusion, the above results indicate that the adipose-derived hMSCs used in this study demonstrated 8% clonogenicity, with about 95% of them expressing all three markers, CD73, CD90 and CD105.

### Temporal expression of RGS2 and RGS4 during adipogenesis and osteogenesis

Our initial interest in members of the regulator of G protein signaling (RGS) family began with a microarray analysis aimed at identifying novel regulators of human adipogenesis (unpublished data). Briefly, the expression profiles of hMSCs exposed to adipogenic differentiation condition (IBMX + DEX + insulin in growth media) was compared to undifferentiated hMSCs at 36 and 72 h post induction. Through this analysis, RGS2 and RGS4 were found to be significantly up- and down-regulated respectively during early adipogenesis, with RGS2 up regulated by 15-folds, and RGS4 down regulated by 100-folds at 72 h post induction. Because adipose and bone cells share common progenitor cells and there are shared regulators between adipogenesis and osteogenesis [17, 18], we expanded our interest in understanding the role of RGS2 and RGS4 in both adipogenesis and osteogenesis.

We first sought to determine the temporal expression pattern of *RGS2* and *RGS4* during both osteogenic and adipogenic differentiation of hMSCs. For adipogenesis, hMSCs were cultured in hyclone growth media (CM) supplemented with IBMX (0.45 µM), DEX (1 µM), and insulin (10 µg/ml), which is abbreviated as adipogenic inducing media (AIM). For osteogenesis, cells were cultured in CM supplemented with DEX (0.2 µM), β-glycerophosphate (10 mM), and ascorbic acid-2-phosphate (0.05 mM), which is abbreviated as osteogenic inducing media (OIM). DEX is a common component used in hMSC differentiation into adipocyte, osteocyte, and chondrocytes, and is thought to be necessary to potentiate differentiation and prevent apoptosis [56, 57]. Therefore, to also dissect the role of individual component of differentiation inducing cocktails on the expression of *RGS2* and *RGS4*, RT-PCR was performed on both genes in hMSCs cultured in 8 different media treatments that include CM (control group), DEX (0.2 µM), DEX (1 µM), DEX (0.2 µM) + IBMX, AIM with 0.2 µM DEX, AIM with 1 µM DEX, OIM with 0.2 µM DEX, and OIM with 1 µM DEX, at eight different time points including D0.5, D1, D1.5, D2, D4, D5, D6 and D7 post initial treatment, with media change at 48-h intervals. All treatment group media was made in CM. Transcript level of *RGS2* and *RGS4* in each treatment group were normalized to that of a housekeeping gene HSP90 (internal control) and compared to its normalized level in CM control at corresponding time point.

**Fig. 1:**
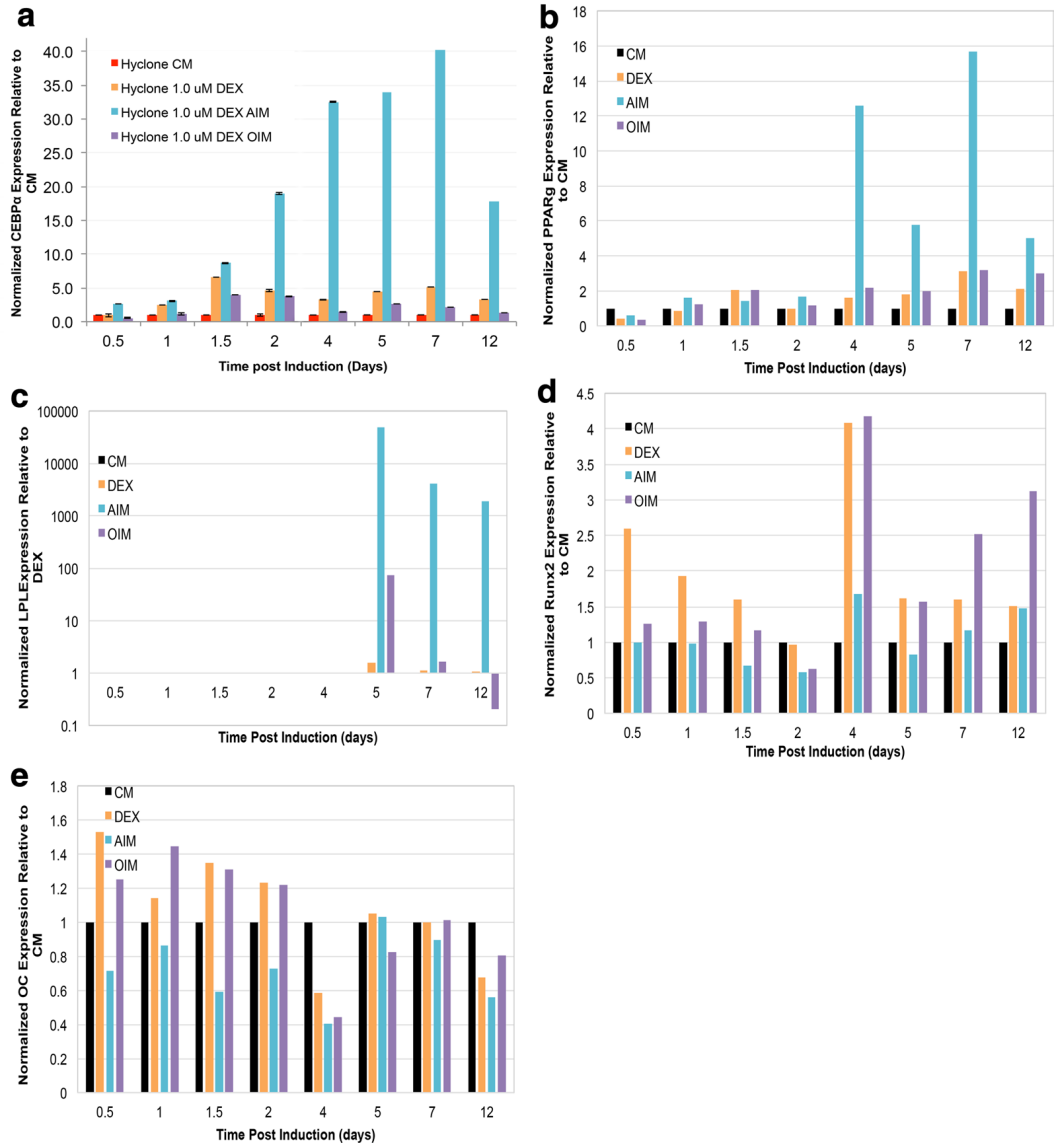
Temporal expression of adipogenic and osteogenic markers in AIM and OIM treatments. Expression of adipogenic markers C/EBPα (**a**), PPARγ (**b**), LPL (**c**) and osteogenic markers Runx2 (**d**) and Osteocalcin (OC) (**e**) in control Hyclone CM, Hyclone CM based 1 µM DEX (Hyclone 1 µM DEX), Hyclone CM based AIM with 1 µM DEX (Hyclone 1 µM DEX AIM), and Hyclone CM based OIM with 1 µM DEX (Hyclone 1 µM DEX OIM) was examined at D0.5, D1, D1.5, D2, D4, D5, D6 and D7 post initial treatment

As expected, expression of *C/EBPα* and *PPARγ*, two well-known master regulators for adipogenic lineage commitment, were both highly up regulated in AIM treated cells (Fig. 1a, b). Similarly, expression of LPL, a relatively later stage adipogenic marker encoding a lipoprotein lipase that breaks down lipids [58]. was also highly enriched in AIM treated cells (Fig. 1c) (Expression of LPL was undetectable in CM groups, so its expression in AIM and OIM treated cells was compared to its expression in DEX treated cells). On the other hand, expression of Runx2, a master regulator for osteogenic differentiation [14], was expressed at a higher level in OIM treated cells relative to AIM treated cells, especially after D4 (Fig. 1d). However, expression of osteocalcin (OC), which encodes a bone specific protein synthesized by osteoblast and serves as a marker of osteogenic maturation [59], appeared to be only slightly upregulated in DEX and OIM treated cells compared to its expression in AIM before D4, but reached to a similar level across all treatment groups thereafter (Fig. 1e).

**Fig. 2:**
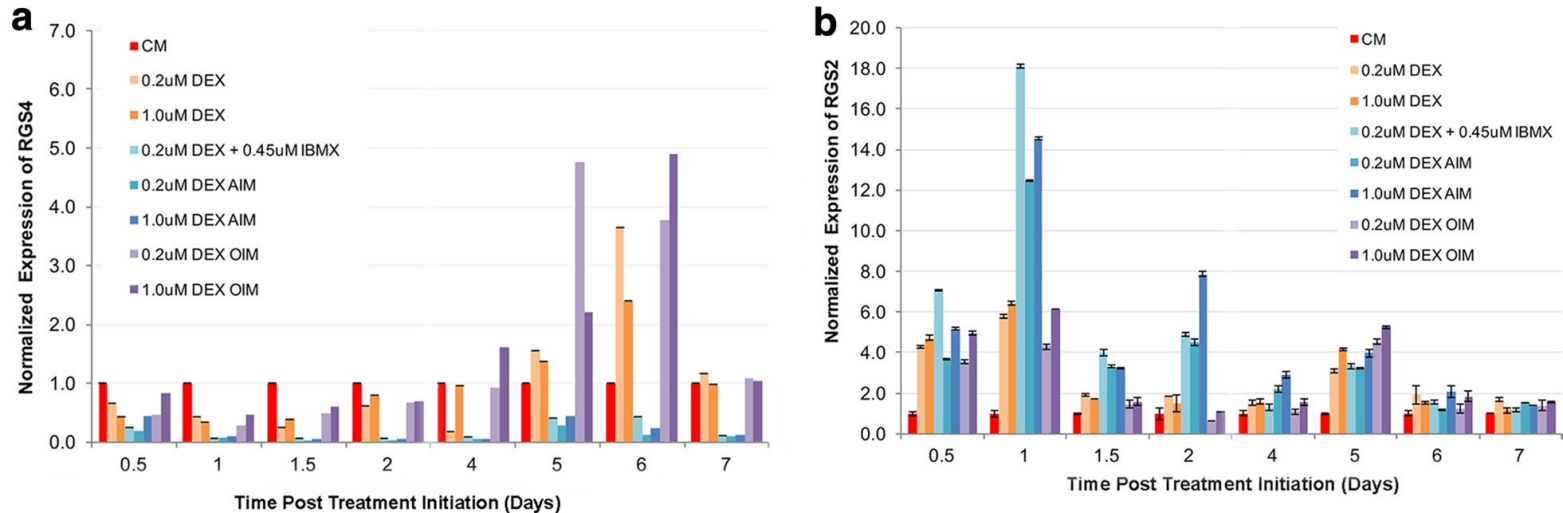
Temporal expression of RGS4 and RGS2 in AIM and OIM treatments. Expression of RGS4 (**a**) and RGS2 (**b**) was examined by RT-PCR in hMSCs cultured in 8 different media treatments, including CM (control), DEX (0.2 µM), DEX (1 µM), DEX (0.2 µM) + IBMX, AIM with 0.2 µM DEX, AIM with 1 µM DEX, OIM with 0.2 µM DEX, and OIM with 1 µM DEX. Expression in each treatment condition was examined at eight different time points, including D0.5, D1, D1.5, D2, D4, D5, D6 and D7 post initial treatment. Graphs represent average gene expression level normalized to that of *HSP90* and set relative to CM control at each given time point (n = 2). **a** Graph of *RGS4* expression. **b** Graph of *RGS2* expression

Expression of *RGS4* remained high in hMSCs cultured in CM throughout D0.5 to D7, but was differentially regulated in response to adipogenic induction (DEX + IBMX or AIM) vs. osteogenic induction (OIM) (Fig. 2a). Starting as early as D0.5, adipogenic induction by DEX + IBMX or AIM, regardless of DEX concentrations, resulted in down regulation of *RGS4* by 2.5- to 5-folds. By D1, *RGS4* was barely detectable and remained significantly down-regulated throughout the remaining treatment duration (Fig. 2). In OIM treatments, again regardless of DEX concentrations, *RGS4* expression followed a similar pattern as in DEX treatment alone, first slightly downregulated by 1.5- to 3.3-folds between D0.5 and D2 and then upregulated by up to 3.75-fold between D4 and D7 (Fig. 2a). Overall, *RGS4* expression is down regulated by all adipogenic treatment conditions starting as early as 12 h post treatment initiation, and upregulated by osteogenic treatments starting D4 in a pattern similar to DEX only treatment.

Expression of *RGS2* is upregulated by all treatment types relative to its expression in control CM, though the degree of changes differs between adipogenic conditions and the other treatment conditions at certain time points (Fig. 2b). In contrast to *RGS4*, *RGS2* expression was very low in hMSCs cultured in CM (Fig. 2b). Similar to *RGS4* however, *RGS2* expression in OIM followed the same pattern as its expression in DEX treatment alone, regardless of DEX concentrations. At D0.5, *RGS2* was upregulated by 3.5- to 5-folds across all treatment groups compared to CM. At D1, *RGS2* expression was induced to 12- to 14-folds higher in all adipogenic treatment conditions as compared to CM, which is about twice its level in DEX alone or OIM conditions. Between D1.5 and D4, its overall expression level was reduced across all treatment groups as compared to D1, but remained about twofold higher in all adipogenic conditions as compared to OIM or DEX alone. By D5, there was no significant difference across different treatment groups and by D6, *RGS2* expression across all treatment groups dropped to similar levels as in CM control. Overall, expression of *RGS2* is upregulated by all treatment types throughout D0.5 to D5, with significantly greater gain in adipogenic conditions (around twofolds) compared to the other treatment conditions between D1 and D4.

In conclusion, expression of both *RGS2* and *RGS4* in OIM treatment was regulated in parallel to that by dexamethasone treatment alone, regardless of DEX concentrations, indicating that the other two components in OIM media, AA-2-P and β-glycerophosphate, had no significant effect on *RGS2* and *RGS4* expression. *RGS2* was upregulated by both DEX and OIM starting as early as D0.5 and subsiding by D6, whereas *RGS4* was slightly downregulated by DEX and OIM during D0.5 to D2 but up-regulated afterwards. On the contrary, expression of both *RGS2* and *RGS4* differs significantly in adipogenic conditions as compared to in DEX alone, regardless of DEX concentration, indicating that IBMX and/or insulin exerted additional effect on their expression. Since their expression in IBMX + DEX inducing media is highly similar as in AIM, it suggests that IBMX, and not insulin, exerts such effect. Similar to DEX treatment, adipogenic treatment enhanced *RGS2* expression until D6, but at significantly higher level (around twofolds) than DEX alone during D1 to D4. For *RGS4* expression however, adipogenic treatment not only significantly inhibited the expression of *RGS4* at a much greater level than DEX alone during D0.5 to D2 (near undetectable level), but also continued to downregulate its expression throughout the remaining course when it was being upregulated by DEX alone. Hence regulation of *RGS2* and *RGS4* expression were completely opposite to each other in adipogenic conditions.

### Regulation of RGS2 and RGS4 during adipogenic and osteogenic differentiation is independent of media type

Since the Hyclone growth media (CM) used in composing the AIM media for adipogenic induction is a proprietary product that might contain unknown growth factor supplement, we wondered whether expression of *RGS2* and *RGS4* would remain similar in AIM based on a different growth media. Temporal expression pattern of *RGS2* and *RGS4* was then re-examined in parallel in culture conditions based on Hyclone CM, heat-inactivated fetal bovine serum (FBS) in DMEM complete media (HI-FBS CM), or FBS in DMEM complete media (FBS CM). The conditions included Hyclone CM or HI-FBS CM, Hyclone CM DEX (1 µM) or HI-FBS CM DEX (1 µM), Hyclone CM based AIM or HI-FBS CM based AIM or FBS CM based AIM (all with 1 µM DEX), and Hyclone CM based OIM (with 1 µM DEX) at D0.5, D1, D1.5, D2, D3 and D4 post adipogenic initiation (Additional file 3: Figure S3).

Similar to previous results, expression of *RGS4* was downregulated by both DEX alone and AIM from D0.5 to D3, regardless whether they were Hyclone CM or HI-FBS CM based, however, its level was significantly higher in Hyclone CM based AIM than in HI-FBS CM based AIM at day 1 and day 2 (Additional file 3: Figure S3A). Similarly, *RGS2* expression was upregulated by both DEX alone and AIM, regardless whether they were Hyclone CM or HI-FBS CM based. However, its surge in Hyclone CM based AIM was significantly greater (by up to twofolds) than that in HI-FBS CM based AIM (Additional file 3: Figure S3B). As a parallel control, expression of *RGS2* and *RGS4* in Hyclone OIM remain similar to previously described results.

In conclusion, the above results indicate that regulation of RGS2 and RGS4 during adipogenic differentiation is independent of media type, though the degree of change could vary. Next, we sought to determine the role of RGS4 and RGS2 during adipogenic and osteogenic differentiation through *siRNA* mediated gene silencing.

### Expression knockdown of RGS2 and RGS4 in differentiating ad-hMSCs by reverse siRNA transfection

Previously, we identified XtremeGENE *siRNA* transfection reagent as a highly efficient *siRNA* delivery system in bone marrow derived hMSCs [17]. To confirm the effectiveness of this transfection reagent in adipose derived hMSCs (Ad-hMSCs), Ad-hMSCs were reverse transfected with *siTOX* or control *siRNA SiCON* at 16.5 nM. The former activates cellular death response while the latter does not target any known genes in the human genome. Total cell numbers at days 1, 2, 6, and 12 post transfection were compared between s*iTOX* and *siCON*. *SiTOX* reduced cell number by 23% (day 1), 84% (day 2), 83% (day 6), and 72% (day 12) compared to *siCON*, without any noticeable cytotoxic effect in *siCON* treated cells (Additional file 4: Figure S4). Based on the above results, future experiments were conducted using 16.5 nM of *siRNA* to achieve 80–90% of transfection efficiency.

**Fig. 3:**
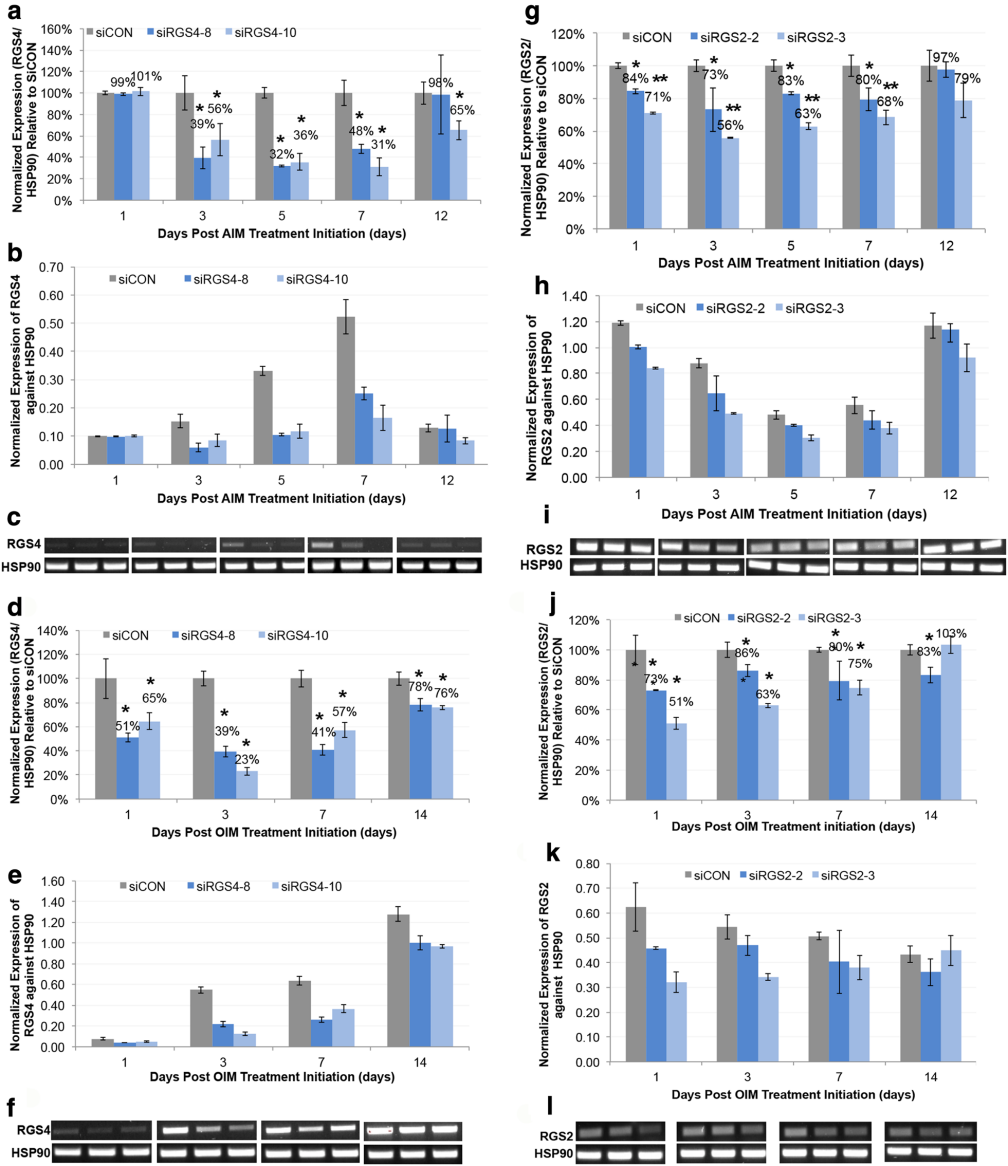
Expression knockdown of RGS4 and RGS2 mRNA induced by siRNA during adipogenic and osteogenic differentiation of hMSCs. Expression of *RGS2* and *RGS4* were examined at day 1, 3, 5, 7, and 12 after differentiation initiation at 48 h post *siRGS2* and si*RGS4* transfection, respectively. **a**–**c** Expression knockdown of *RGS4* by *siRGS4*-*8* and *siRGS4*-*10* during adipogenic differentiation of hMSCs. **d**–**f** Expression knockdown of *RGS4* by *siRGS4*-*8* and *siRGS4*-*10* during osteogenic differentiation of hMSCs. **g**–**i** Expression knockdown of *RGS2* by *siRGS2*-*2* and *siRGS2*-*3* during adipogenic differentiation of hMSCs. **j**–**l** Expression knockdown of *RGS2* by *siRGS2*-*2* and *siRGS2*-*3* during osteogenic differentiation of hMSCs. In **a**, **d**, **g** and **j**, expression level of *RGS2* or *RGS4* in each treatment group was determined relative to their expression in *siCON* control group, after normalization against the expression level of internal control *HSP90* at each given time point. In **b**, **e**, **h** and **k**, expression level of *RGS2* or *RGS4* in each treatment group was normalized against the expression level of internal control HSP90 at each given time point. In **c**, **f**, **i** and **l**, agarose gel images of *RGS4* or *RGS2* and HSP90 RT-PCR products were shown. Error bars represent variation between independent repeats (n = 2). Expression comparison was made between *siCON* and *siRGS4* or *siRGS2* treatment groups at each time point. *p < 0.05, **p < 0.01

To examine the role of RGS2 and RGS4 in ad-hMSCs differentiation into adipocytes and osteocytes, *siRNAs* commercially validated against two different regions of the *RGS4 mRNA* (*siRGS4*-*8* and *siRGS4*-*10*) and *RGS2 mRNA* (*siRGS2*-*2* and *siRGS2*-*3*) were tested. *SiRNA* was transfected into cells at 2 days (D-2) prior to adipogenic differentiation initiation (D0 AIM). Expression of *RGS2* and *RGS4* in transfected ad-hMSCs were examined at day 1, 3, 5, 7, and 12 post adipogenic initiation with AIM containing 1.0 µM DEX or osteogenic initiation with OIM containing 0.2 µM DEX (Fig. 3). Expression level of *RGS2* and *RGS4* in each treatment group was normalized against the expression level of HSP90 and then graphed relative to its normalized expression in *siCON* control group at the same time point.

In samples treated with AIM, *RGS4* mRNA was significantly (p < 0.05) lower at day 3 (39%), 5 (32%), and 7 (48%) in *siRGS4*-*8* treatment groups compared to *siCON* controls (100%) (Fig. 3a, b). Similarly, in *siRGS4*-*10* treatment groups, *RGS4 mRNA* was also significantly (p < 0.05) lower at day 3 (56%), 5 (36%), 7 (31%) and 12 (65%) compared to controls (100%) (Fig. 3a, b). In samples treated with OIM, expression of *RGS4* was significantly (p < 0.05) lower in *siRGS4*-*8* samples at day 1 (51%), 3 (39%), 7 (41%) and 12 (78%) compared to *siCON* (100%) (Fig. 3c, d). Likewise, in *siRGS4*-*10* treatments, *RGS4 mRNA* was significantly (p < 0.05) lower at day 1 (65%), and 3 (23%), 7 (57%) and 14 (76%) (Fig. 3c, d). Overall, *siRGS4* resulted in around 50–75% expression knockdown in *RGS4* expression at the RNA level and there was no significant difference between *siRGS4*-*8* and *siRGS4*-*10* in *RGS4* expression knockdown at any time point tested during adipogenesis or osteogenesis.

To examine the expression knockdown of RGS4 at the protein level, western blots were carried out using two types of antibodies that recognize two different motifs of RGS4 protein separately (see “Methods”). One binds to the C-terminal sequence (amino acids 182–205) outside of the RGS domain (amino acids 62–178) and detects the RGS4 isoform 3 product at 34 kDa. The other binds to the N-terminal sequences (amino acids 40–82) and detects the RGS4 isoforms 1 and 2 both at about 23 kDa. Expression level of RGS4 was compared between *siRGS4*-*8* and *siRGS4*-*10* transfected cells and *siCON* transfected cells at day 1, 2, 3, 4, 5 and 7 post AIM or OIM treatment initiation. Expression level remained similar among all groups at all time points examined (data not shown), except for day 7, when expression of RGS4 isoform 3 was consistently, thought slightly, down regulated in *siRGS4*-*8* and *siRGS4*-*10* transfected cells as compared to in *siCON* transfected cells by about 30 and 20% in AIM and OIM treatment condition, respectively (expression in OIM is shown in Additional file 5: Figure S5A). This delayed and subtle change at the protein level may imply much greater RGS4 protein stability as compared to its *RNA* transcript, in addition to other plausible causes (see “Discussion”).

In samples treated with AIM, *RGS2 mRNA* was significantly (p < 0.05) lower in s*iRGS2*-*2* samples at day 1 (84%), 3 (73%), 5 (83%), and 7 (80%) compared to *siCON* (100%) (Fig. 3e, f). The effect of *siRGS2*-*2* was gone by day 12. Similarly, in *siRGS2*-*3* treatments, *RGS2 mRNA* was significantly (p < 0.01) lower at day 1 (71%), and 3 (56%), 5 (63%), 7 (68%) and 12 (79%) (Fig. 3e, f). In samples treated with OIM, *RGS2 mRNA* was significantly (p < 0.05) lower in *siRGS2*-*2* samples at day 1 (73%), and non-significantly lower by day 3 (86%), 7 (80%), and 12 (83%) (Fig. 3g, h). In *siRGS2*-*3* samples, *RGS2 mRNA* was significantly (p < 0.05) down regulated at day 1 (51%), day 3 (63%), day 7 (75%), but resumed to control level by day 12 (103%) (Fig. 3g, h). Overall, *siRGS2*-*2* and *siRGS2*-*3* resulted in expression knockdown of *RGS2* by 20–30 and 30–50%, respectively.

Since upregulation of *RGS2* expression upon adipogenic initiation was significantly greater in Hyclone CM based AIM as compared to HI-FBS CM based AIM, and considering that the expression knockdown by *siRGS2* was modest in Hyclone CM based AIM (by 20–50%), we wondered whether *siRGS2* would have greater knockdown in HI-FBS CM based AIM due to lower basal level of *RGS2* expression, and hence greater phenotypic effect. Similar to previous studies, expression knockdown of *RGS2* by *siRGS2*-*2* and *siRGS2*-*3* was evaluated at days 1, 3, 5, 7, and 14 post treatment initiation with HI-FBS CM based AIM at 48 h after *siRNA* transfection. In *siRGS2*-*2* samples, expression of *RGS2* was significantly lower (p < 0.05) at day 3 (69%), 5 (60%) and 7 (51%), but resumed to control levels (97%) at day 12 as compared to *siCON* samples (100%) (Additional file 6: Figure S6). Similarly, in *siRGS2*-*3* samples, expression of *RGS2* was significantly lower (p < 0.05) at day 1 (43%), 3 (34%), 5 (32%), and 7 (49%) and resumed to control level by day 12 (94%) compared to *siCON* (100%) (Additional file 6: Figure S6).

Expression knockdown of RGS2 at the protein level was also examined by western blots in *siRGS2*-*2*, *siRGS2*-*3* or *siCON* treated cells under AIM and OIM treatment conditions using Hyclone CM based media. Consistently, expression of RGS2 was down regulated by 30–40% in *siRGS2*-*2* cells and 60–70% in *siRGS2*-*3* cells as compared to in *siCON* cells on day 2 post AIM or OIM treatment initiation (expression in OIM is shown in Additional file 5: Figure S5B), which correlates well to the level of expression knockdown detected at the *RNA* level as shown above.

In conclusion, during early adipogenic and osteogenic treatments in Hyclone CM based media, both *siRGS4*-*8* and *siRGS4*-*10* downregulated *RGS4* expression by about 50–75% at the *RNA* level, but only about 20–30% expression knockdown was detected in isoform 3 of RGS4 at the protein level, whereas *siRGS2*-*2* and *siRGS2*-*3* downregulated *RGS2* expression by 20–30 and 30–50% respectively at the *RNA* level and similarly by 30–40 and 60–70% respectively at the protein level. Additionally, in HI FBS CM based AIM condition, *siRGS2*-*3* exerted a greater level of gene silencing (by 50–70%) compared to *siRGS2*-*2* (by 30–50%) at the *RNA* level, both of which are greater than their respective silencing effect in Hyclone CM based AIM. Next, we examined the effect of expression knockdown induced by *siRGS2 and siRGS4* on adipogenic and osteogenic differentiation of hMSCs.

### Expression knockdown of RGS2 and RGS4 exerts different levels of inhibitory effect on adipogenic differentiation of ad-hMSCs

To investigate the role of RGS2 and RGS4 in adipogenesis, we measured the effect of their expression knockdown induced by *siRNA* on several metrics of adipogenesis. As described previously, ad-hMSCs were reverse transfected with 16.5 nM of control (*siCON*) or targeted *siRNA* in Hyclone growth media (CM). After 48 h, adipogenesis was initiated by AIM with 1.0 μM DEX. After 12 days of AIM treatment, with media change at 48-h intervals, cells were fixed and stained with DAPI (nuclear stain) and OilRedO (oil droplet staining). Overlapping images of DAPI and OilRedO stained cells were taken from multiple wells of each treatment group for total cell counting, adipocytes counting and area measurements of stained lipid droplets in OilRedO images, and OilRedO dye was subsequently extracted with isopropanol and quantified by absorbance reading at 515 nm (see “Methods”).

**Fig. 4:**
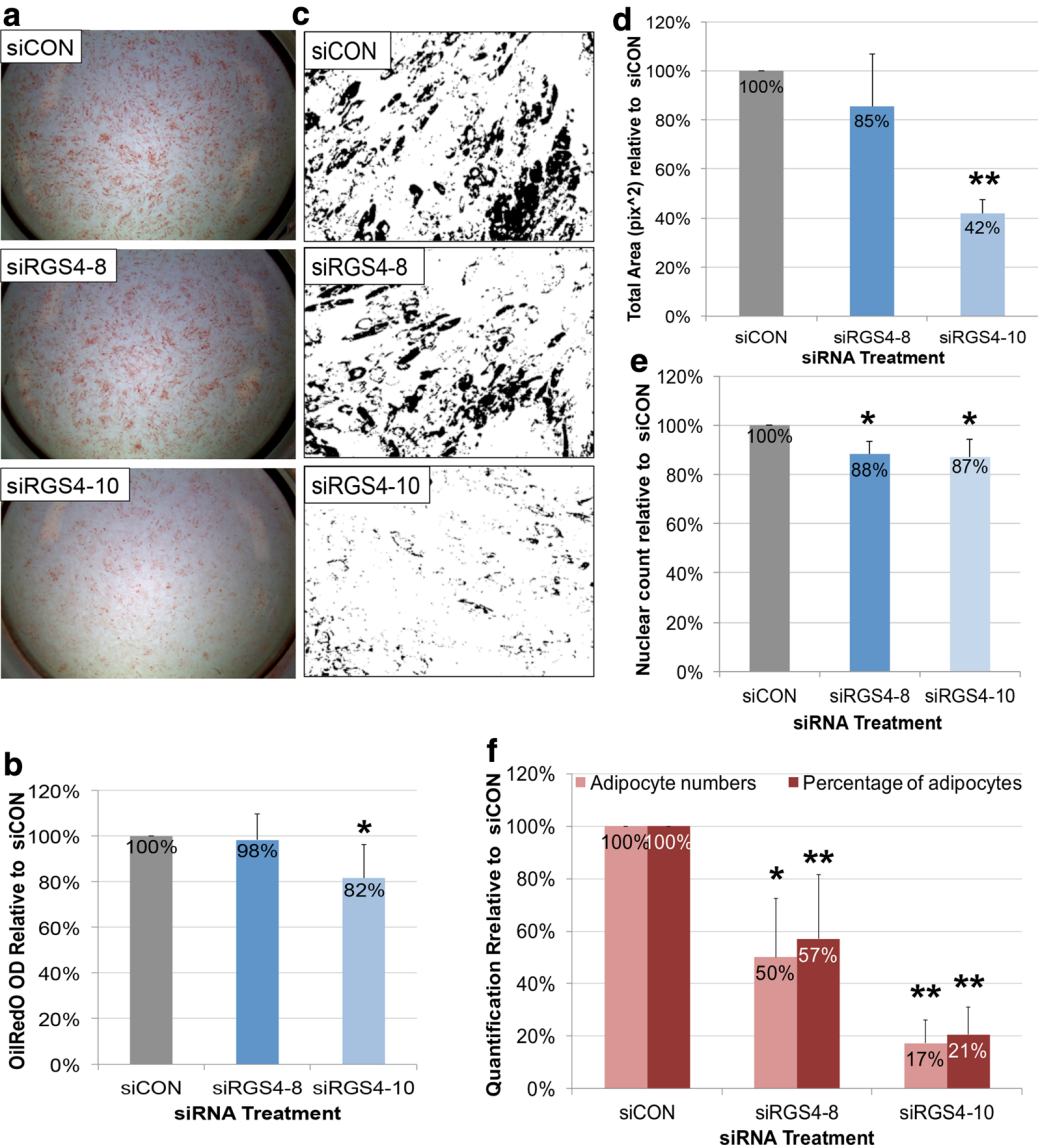
Effect of siRGS4 on adipogenic differentiation of hMSCs induced by Hyclone CM based adipogenic media. **a** Phase contrast images of OilRedO stained wells at day 12 post adipogenic initiation. Lipid droplets were stained red. **b** OilRedO staining quantification by absorbance reading at 515 nm. **c** Representative ImagePro area measurement images showing positively stained oil droplets in black and unstained cells in white. **d** Area measurement quantification of stained oil droplets. **e** Quantification of total nuclear count per treatment group. **f** Quantification of adipocyte count and percentage of adipocyte per treatment group. Images and graphs represent the mean quantification of *siRGS4* treatment wells set relative to that of *siCON* treatment wells from a representative experimental (n = 3). Comparison was made between *siCON* and *siRGS4* treatment groups. *p < 0.05, **p < 0.01

For *RGS4* expression knockdown, whole-well images showed noticeably lower intensity of OilRedO stains in *siRGS4*-*10* but not *siRGS4*-*8* groups compared to *siCON* control (Fig. 4a). Correspondingly, OilRedO quantification was significantly lower in *siRGS4*-*10* treatment group (82%, p < 0.05) compared to *siCON* controls (100%), and the difference between *siRGS4*-*8* treatment group (98%) and *siCON* control (100%) was insignificant (Fig. 4b). Consistently, area measurements of stained oil droplets were significantly lower in *siRGS4*-*10* treatment groups (42%, p < 0.01) compared to *siCON* (100%), but insignificantly lower in *RGS4*-*8* (85%) compared to *siCON* (100%) (Fig. 4c, d). Differences in total fat accumulation could be a result of variation in adipocyte numbers and/or variation in lipid accumulation within individual adipocytes. To determine the cause, total cell counts and adipocyte cell counts were determined based on DAPI nuclear stain and manual identification of mature adipocytes in OiRedO images respectively. Total cell numbers were significantly lower in both *siRGS4*-*8* (88%, p ≤ 0.05) and *siRGS4*-*10* (87%, p ≤ 0.05) treatment groups compared to *siCON* controls (100%) (Fig. 4e). Adipocyte cell numbers were even more drastically lower in both *siRGS4*-*8* (50%, p < 0.05) and *RGS4*-*10* (17%, p < 0.01) treatment groups compared to *siCON* controls (100%) (Fig. 4f). Percentage of adipocytes calculated by adipocytes number/total cell number was also significantly lower in *siRGS4*-*8* (57%, p < 0.01) and *siRGS4*-*10* (21%, p < 0.01) treatment groups compared to *siCON* controls (100%) (Fig. 4f). Overall, expression knockdown of *RGS4* by *siRGS4* resulted in significantly decreased total fat accumulation, total cell numbers, total adipocyte numbers and differentiation efficiency as reflected by percentage of adipocytes, with *siRGS4*-*10* exerting greater effect than *siRGS4*-*8*.

**Fig. 5:**
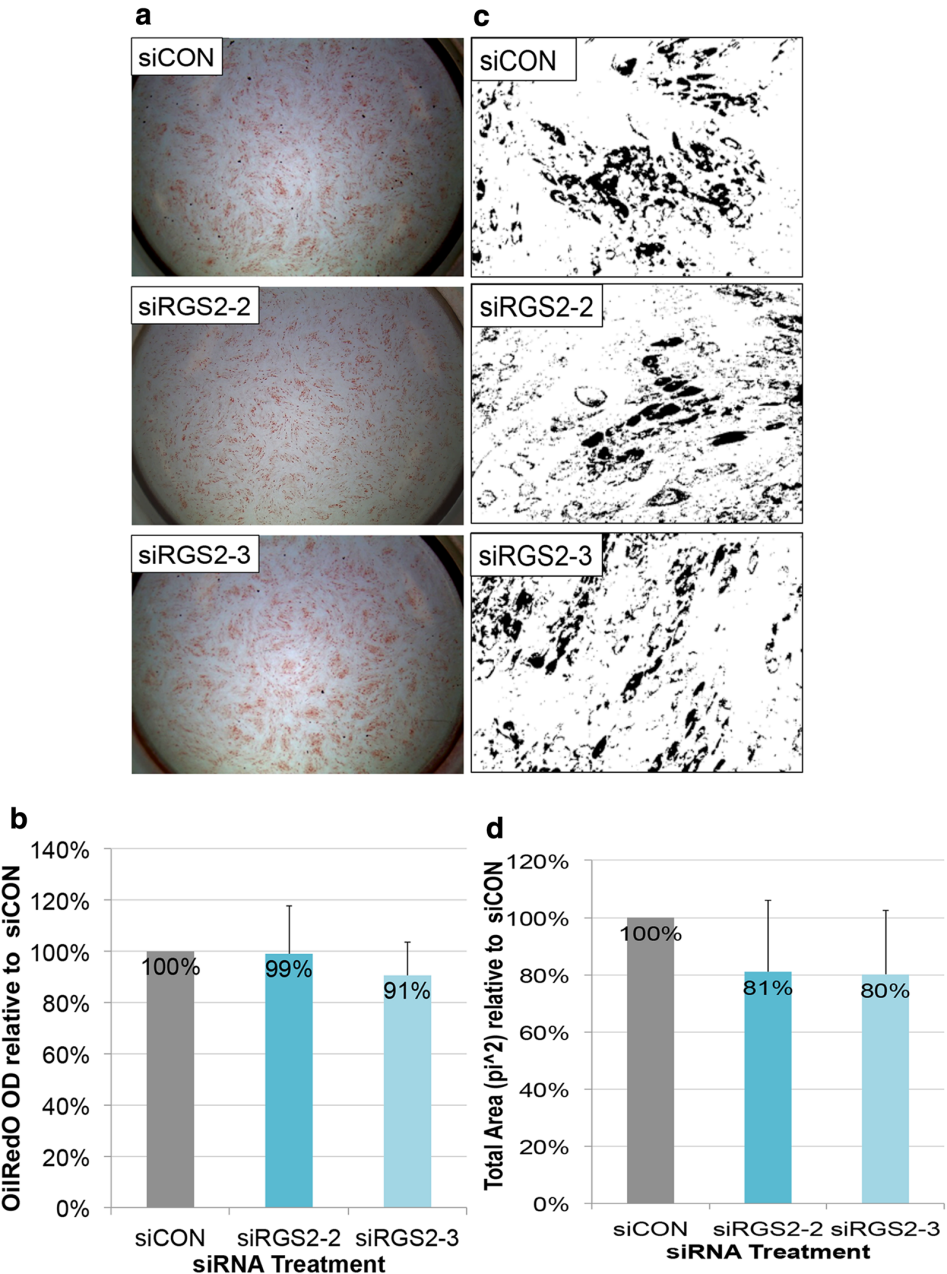
Effect of siRGS2 on adipogenic differentiation of hMSCs induced by Hyclone CM based adipogenic media. **a** Phase contrast images of OilRedO stained wells at day 12 post adipogenic initiation. Lipid droplets were stained red. **b** OilRedO staining quantification by absorbance reading at 515 nm. **c** Representative ImagePro area measurement images showing positively stained oil droplets in black and unstained cells in white. **d** Area measurement quantification of stained OilRedO oil droplets. Images and graphs represent the mean quantification of *siRGS2* treatment wells set relative to that of *siCON* treatment wells from a representative experimental set (n = 3)

For *RGS2* expression knockdown, there was no noticeable difference in OilRedO staining intensity (Fig. 5a). OilRedO quantification was not significantly different between *siRGS2*-*2* (99%) or *siRGS2*-*3* (91%) treatment groups and *siCON* controls (100%) neither (Fig. 5b). Consistently, total area (pi^2^) measurements of OilRedO stained oil droplets trended lower in both *siRG2*-*2* (81%) and *siRGS2*-*3* (80%) treatment groups compared to *siCON* controls (100%), but the difference was not statistically significant (Fig. 5c, d). Nuclear counts in *siRGS2*-*2* (96%) and *siRGS2*-*3* (97%) slightly but consistently trended lower than *siCON* controls (100%), though statistically insignificant neither (data not shown). Overall, *siRGS2* did not significantly affect total fat areas or total cell numbers as compared to *siCON* treatment in Hyclone CM based AIM condition.

**Fig. 6:**
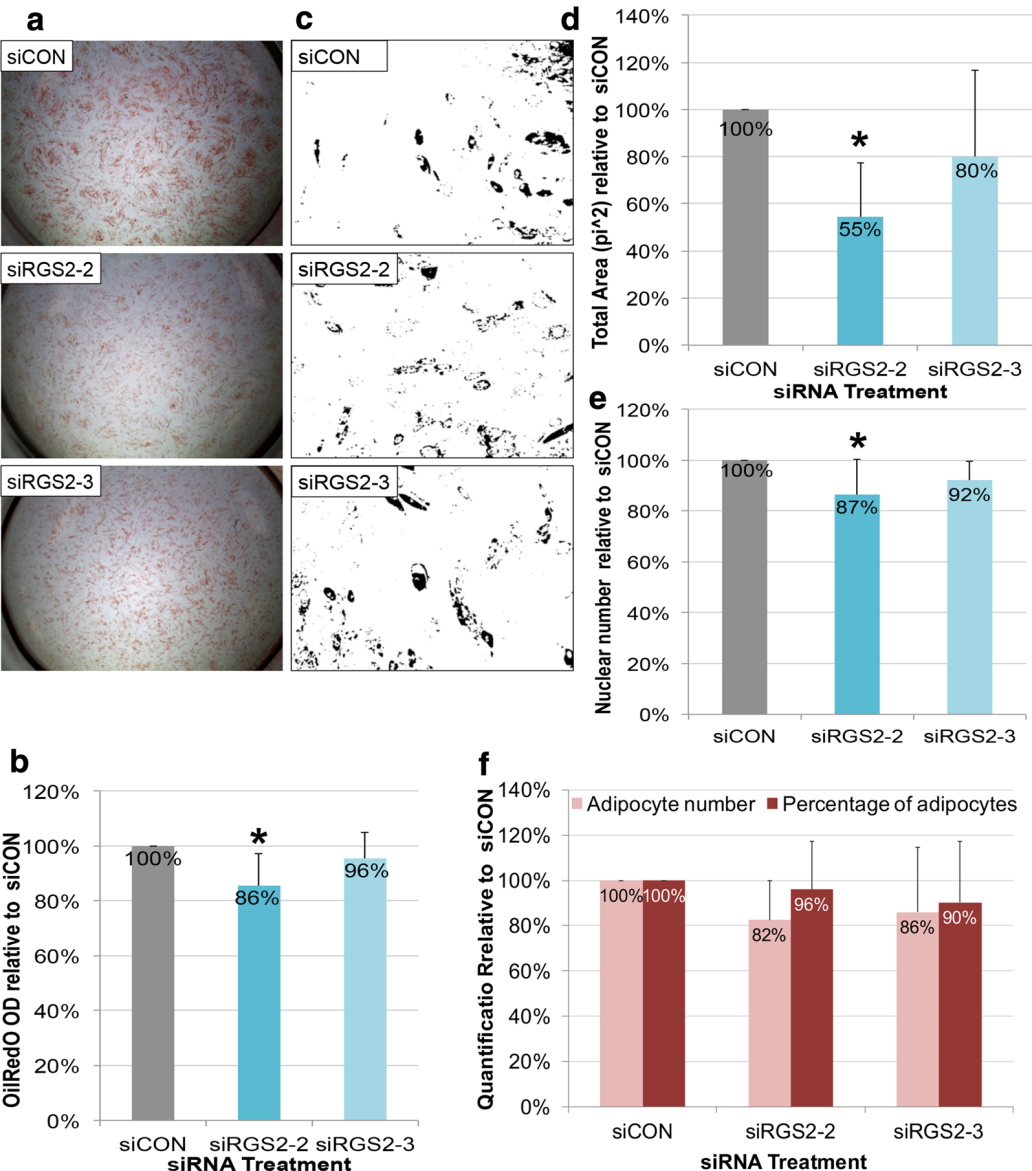
Effect of siRGS2 on adipogenic differentiation of hMSCs induced by HI-FBS CM based AIM. Ad-hMSCs were transfected 2 days prior to differentiation induction with HI-FBS CM based AIM media. **a** Phase contrast images of OilRedO stained wells at day 12 post adipogenic initiation. Lipid droplets were stained red. **b** OilRedO staining quantification by absorbance reading at 515 nm. **c** Representative ImagePro area measurement images showing positively stained oil droplets in black and unstained cells in white. **d** Area measurement quantification of stained oil droplets. **e** Total cell count based on DAPI nuclear stain. **f** Adipocytes cell count and percentage of adipocytes. Images and graphs represent the mean quantification of *siRGS4* treatment wells set relative to that of *siCON* wells from a representative experimental set (n = 3). Comparison was made between *siCON* and *siRGS2* treatment groups. *p < 0.05

The effect of *siRGS2* on adipogenesis induced by HI FBS CM based AIM was also analyzed. In contrary to Hyclone CM based AIM condition, OilRedO stain intensity in both *siRGS2*-*2* and *siRGS2*-*3* treatments appeared visually reduced compared to *siCON* (Fig. 6a). Total fat accumulation quantification by OilRedO dye extraction however was only significantly lower in *siRGS2*-*2* (86%, p < 0.05) but not in *siRGS2*-*3* (96%) treatment groups as compared to *siCON* controls (100%) (Fig. 6b). Consistently, total area measurements (pi^2^) of stained oil droplets was significantly lower in *siRGS2*-*2* (55%, p < 0.05) and only trended lower in *siRGS2*-*3* (80%, p < 0.1) treatments as compared to in *siCON* controls (100%) (Fig. 6c, d). Subsequently we determined whether decreased total fat accumulation was the result of reduction in adipocyte numbers and/or differentiation efficiency. Total nuclear counts were only significantly lower in *siRGS2*-*2* (87%, p < 0.05) but not in *siRGS2*-*3* (92%) treatment groups compared to *siCON* controls (100%) (Fig. 6e). Total adipocyte number trended lower in both *siRGS2*-*2* (82%, p < 0.1) and *siRGS2*-*3* (86%) treatments compared to *siCON* controls (100%) (Fig. 6f). Percent of adipocytes in *siRGS2*-*2* (96%) and *siRGS2*-*3* (90%) groups were not significantly different from *siCON* controls (100%) (Fig. 6f). Overall, in the HI FBS CM based AIM induced adipogenic differentiation, *siRGS2*-*3* had mild inhibitory effect that was statistically deemed insignificant, but *siRGS2*-*2* treatment significantly inhibited total fat accumulation as compared to *siCON*, which was likely the consequence of significantly reduced total number of cells, as differentiation efficiency determined by percentage of adipocytes was not significantly different between *siRGS2*-*2* and *siCON*.

In conclusion, expression knockdown of *RGS4* by 50–75% significantly inhibited adipogenic differentiation of hMSCs by reducing total adipocytes and adipogenic differentiation efficiency, with *siRGS4*-*10* exerting greater effect than *siRGS4*-*8*. Expression knockdown of *RGS2* also exhibited similar inhibitory effect in HI-FBS CM based AIM but not Hyclone CM based AIM conditions, likely due to greater expression knockdown in the former vs. the latter, with *siRGS2*-*2* exerting greater effect than *siRGS2*-*3*. Such effect was at least partly due to reduced total adipocytes as the result of reduced total cell numbers, without affecting differentiation efficiency.

### Effects of siRGS2 and siRGS4 on the expression of adipogenic markers

Expression knockdown of *RGS4* by *siRGS4* resulted in significantly decreased adipogenesis in part due to reduced total cell numbers. Since differentiation efficiency as reflected by percentage of adipocytes was also reduced, it indicated that decreased adipocyte number in *siRGS4* treatment groups might not be solely due to reduction in total cell number and *siRGS4* might affect adipogenesis directly, resulting in decreased differentiation efficiency. On the other hand, *siRGS2* had insignificant effect on adipogenesis in response to Hyclone CM based AIM but significant inhibitory effect on adipogenesis in response to HI FBS CM based AIM, without significantly affecting adipogenic differentiation efficiency. This indicated that siRGS2 did not likely affect adipogenesis directly. To test the above, the effect of *siRGS2* or *siRGS4* on the expression of selected adipogenic makers, *PPARγ*, *C/EBPα* and *LPL*, was measured at day 1, 3, 5, 7, and 12 post adipogenic induction (Hyclone CM based AIM) in hMSCs that had been subjected to *siRGS2*/*siRGS4* or *siCON* transfection. Similar to previous expression analyses, expression of those genes in *siRGS2/siRGS4* treated cells was measured by RT-PCR and compared to its value in *siCON* treated samples at the same time point, after normalization to the expression level of internal control *HSP90*.

**Fig. 7:**
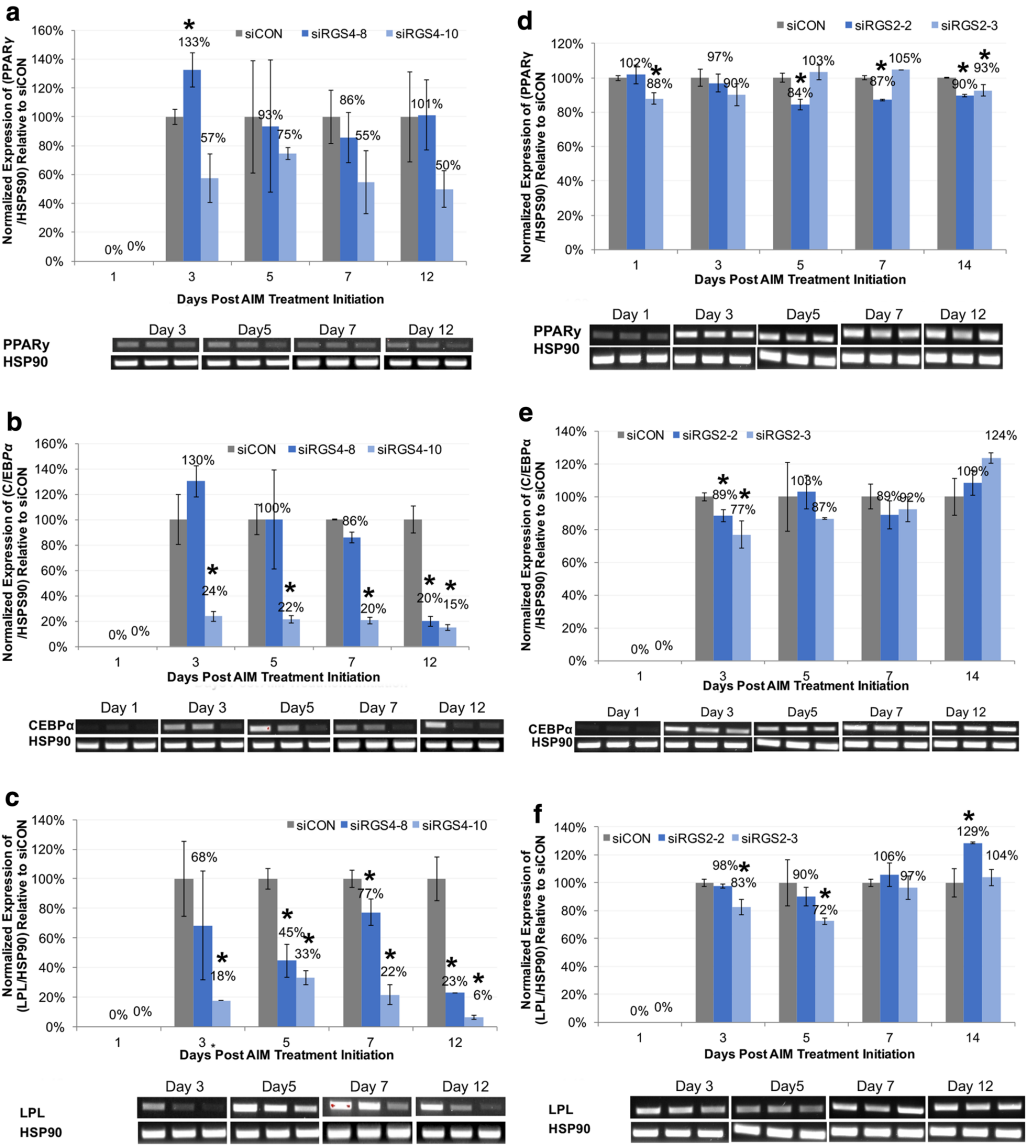
Effect of siRGS4 and siRGS2 on the expression of adipogenic markers during adipogenic differentiation of hMSCs induced by Hyclone CM based adipogenic media. Expression of *PPARγ*, *C/EBPα* and *LPL* were examined at day 1, 3, 5, 7 and 12 post adipogenic treatment initiation in *siCON* and *siRGS4* (**a**–**c**) or *siRGS2* (**d**–**f**) transfected AD-hMSCs. Graphs represent average expression level of each gene normalized to that of *HSP90* and set relative to its normalized expression in *siCON* transfected cells. Agarose gels show RT-PCR products of examined genes and *HSP90* at indicated time points. **a** Expression of *PPARγ* in *siRGS4* and *siCON* transfected cells. **b** Expression of *C/EBPα* in *siRGS4* and *siCON* transfected cells. **c** Expression of *LPL* in *siRGS4* and *siCON* transfected cells. **d** Expression of *PPARγ* in *siRGS2* and *siCON* transfected cells. **e** Expression of *C/EBPα* in *siRGS2* and *siCON* transfected cells. **f** Expression of *LPL* in *siRGS2* and *siCON* transfected cells. Error bars represent variation between independent repeats (n = 2). Expression comparison was made between *siCON* and *siRGS2* or *siRGS4* treatment groups at each given time point. *p < 0.05, **p < 0.01

In *siRGS4* samples, expression levels of *PPARγ* and *C/EBPα* in *siRGS4*-*8* were not significantly different from *siCON* controls at all time points examined, except for *PPARγ* upregulation at day 1 (130%, p < 0.05) and *C/EBPα* downregulation at day 12 (20%, p < 0.05) compared to *siCON* (100%) (Fig. 7a, b). However, in *siRGS4*-*10* samples *PPARγ* expression level overall trended lower while *C/EBPα* was significantly down regulated at all time points (15–25%, p < 0.05) compared to *siCON* controls (100%) (Fig. 7a, b). Expression of *LPL* on the other hand was significantly down regulated in both *siRGS4*-*8* and *siRGS4*-*10*, with 45% at day 5, 77% at day 7 and 23% at day 12 in the former and 18% at day 3, 33% at day 5, 22% at day 7 and 6% at day 12 in the latter samples as compared to *siCON* (100%) (Fig. 7c). Overall, expression of both *C/EBPα* and *LPL* were significantly down regulated by *siRGS4*-*10* at multiple time points during adipogenic differentiation, whereas only *LPL* was down regulated by *siRGS4*-*8* at multiple time points, which is consistent with the more disruptive effect of *siRGS4*-*10* on adipogenic differentiation of hMSCs as compared to *siRGS4*-*8*.

In *siRGS2* samples, expression level of *PPARγ* was slightly but significantly lower in *siRGS2*-2 treatments (84–90%) as compared to *siCON* (100%) on day 5, 7 and 12, and similar difference between *siRGS2*-*3* and *siCON* was observed on day 1 and day 12 (Fig. 7e). Expression of *C/EBPα* was also slightly but significantly downregulated by *siRGS2*-*2* (89%) and *siRGS2*-*3* (77%) on day 3 compared to *siCON* (100%), but remained unchanged at the other time points (Fig. 7f). Expression of *LPL* was upregulated in *siRGS2*-*2* samples at day 12 (129%, p < 0.05) compared to *siCON* controls (100%), but was not significantly changed at the other time points. In *siRGS2*-*3* samples, levels of *LPL* were slightly but significantly lower at day 3 (83%) and 5 (72%) compared to *siCON* samples (100%), but remained insignificantly different at the other time points (Fig. 7g). Overall, both *siRGS2*-*2* and *siRGS2*-*3* had a subtle suppressive effect on the expression of both *PPARγ* and *C/EBPα*, and only *siRGS2*-3 appeared to have a subtle suppressive effect on the expression of *LPL*, consistent with the overall mild and insignificant effect of *siRGS2* on total fat accumulation in Hyclone CM based AIM condition.

**Fig. 8:**
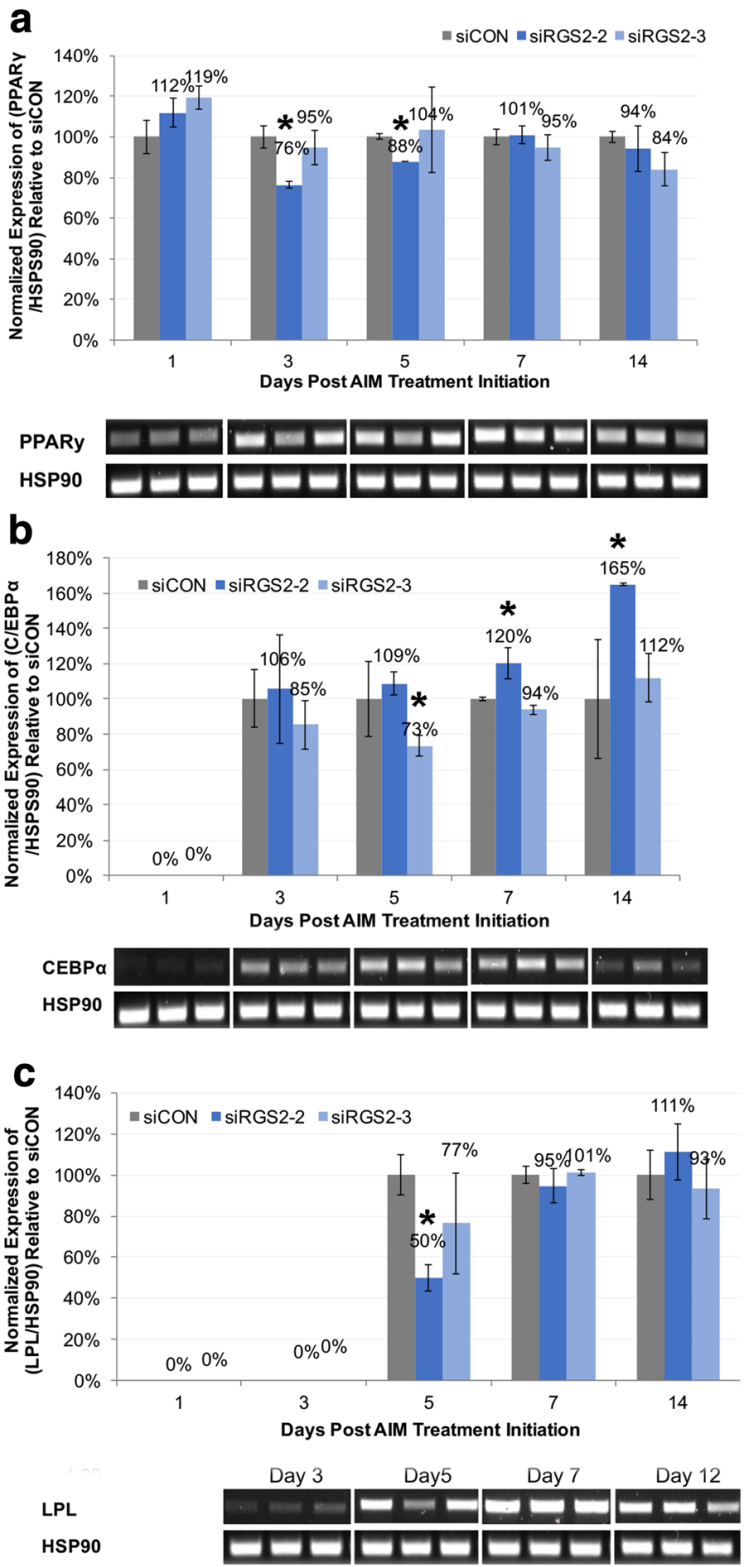
Effect of siRGS2 on the expression of adipogenic markers during adipogenic differentiation of hMSCs induced by HI FBS CM based adipogenic media. Expression of *PPARγ*, *C/EBPα* and *LPL* were examined at day 1, 3, 5, 7 and 12 post adipogenic treatment initiation in *siCON* and *siRGS2* transfected AD-hMSCs. Graphs represent average expression level of each gene normalized to that of *HSP90* and set relative to its normalized expression in *siCON* transfected cells. Agarose gels show RT-PCR products of examined genes and *HSP90* at indicated time points. **a** Expression of *PPARγ* in *siRGS2* and *siCON* transfected cells. **b** Expression of *C/EBPα* in *siRG2* and *siCON* transfected cells. **c** Expression of *LPL* in *siRGS2* and *siCON* transfected cells. Error bars represent variation between independent repeats (n = 2). Expression comparison was made between *siCON* and *siRGS2* or *siRGS4* treatment groups at each given time point. *p < 0.05

Since *siRGS2* exerted significant inhibitory effect on adipogenic differentiation of hMSCs induced by HI-FBS CM based AIM, expression of all four adipogenic marker genes was also examined in such condition. Expression of *PPARγ* was slightly but significantly down regulated by *siRGS2*-*2* at day 3 (76%, p < 0.05) and day 5 (88%, p < 0.05) but not by *siRGS2*-*3* as compared to *siCON* (100%) (Fig. 8a). *C/EBPα* expression on the other hand was upregulated in *siRGS2*-*2* treatment groups at day 7 (120%, p < 0.05) and 12 (160%, p < 0.05), but slightly downregulated in *siRGS2*-*3* at day 3 (73%, p < 0.05) compared to *siCON* (100%) (Fig. 8b). *LPL* expression was only downregulated by *siRGS2*-*2* (50%, p < 0.05) at day 5 but not by *siRGS2*-*3* compared to *siCON* (100%) (Fig. 8c). Overall, *siRGS2*-*2* slightly down regulated expression of *PPARγ* and *LPL*, but upregulated *C/EBPα*, whereas *siRGS2*-*3* had minimum effect on the expression of these genes except for transient downregulation of *C/EBPα*. This is consistent with previous observation that compared to *siRGS2*-*3*, *siRGS2*-*2* exerted greater inhibitory effect on adipogenesis induced by HI FBS CM based AIM. In addition, effect of *siRGS2*-*2* on adipogenic gene expression was only slight, consistent with previous observation that *siRGS2*-*2* did not significantly affect differentiation efficiency determined by percentage of adipocytes, and its inhibitory effect on adipogenesis was mainly likely due to reduced adipocytes as the result of reduced total cell numbers and possibly reduced fat accumulation per adipocyte as well.

In conclusion, consistent with their different levels of inhibitory effect on the adipogenic outcome of hMSCs, *siRGS4* exerted significantly greater level of inhibition on the expression of adipogenic marker genes (*PPARγ*, *C/EBPα*, and *LPL*) than *siRGS2*. In addition, *siRGS4*-*10* downregulated all three genes whereas *siRGS4*-*8* only inhibited *LPL*, which is also consistent with the more disruptive effect of *siRGS4*-*10* on adipogenic differentiation of hMSCs as compared to *siRGS4*-*8*.

### Effect of siRGS2 and siRGS4 in osteogenic differentiation of hMSCs

To investigate the potential role of RGS2 and RGS4 during osteogenic differentiation of hMSCs, we applied the same D-2/D0 *siRNA* transfection approach. Briefly, even number of ad-hMSCs were reverse transfected with xtremeGENE/*siRNA* complex at 16.5 nM in Hyclone control (CM) media. After 48 h, osteogenesis was induced by 0.2 μM DEX OIM media, which was subsequently changed every 48 h. After 18–26 days of OIM media treatment, cells were fixed and stained with alizarin red S, which specifically stains for calcific deposit (hydroxylapatite) by osteocytes. Alizarin Red S dye was subsequently extracted with acetic acid and quantified calorimetrically at 405 nm as a measurement of osteogenic differentiation efficiency (see “Methods”).

**Fig. 9:**
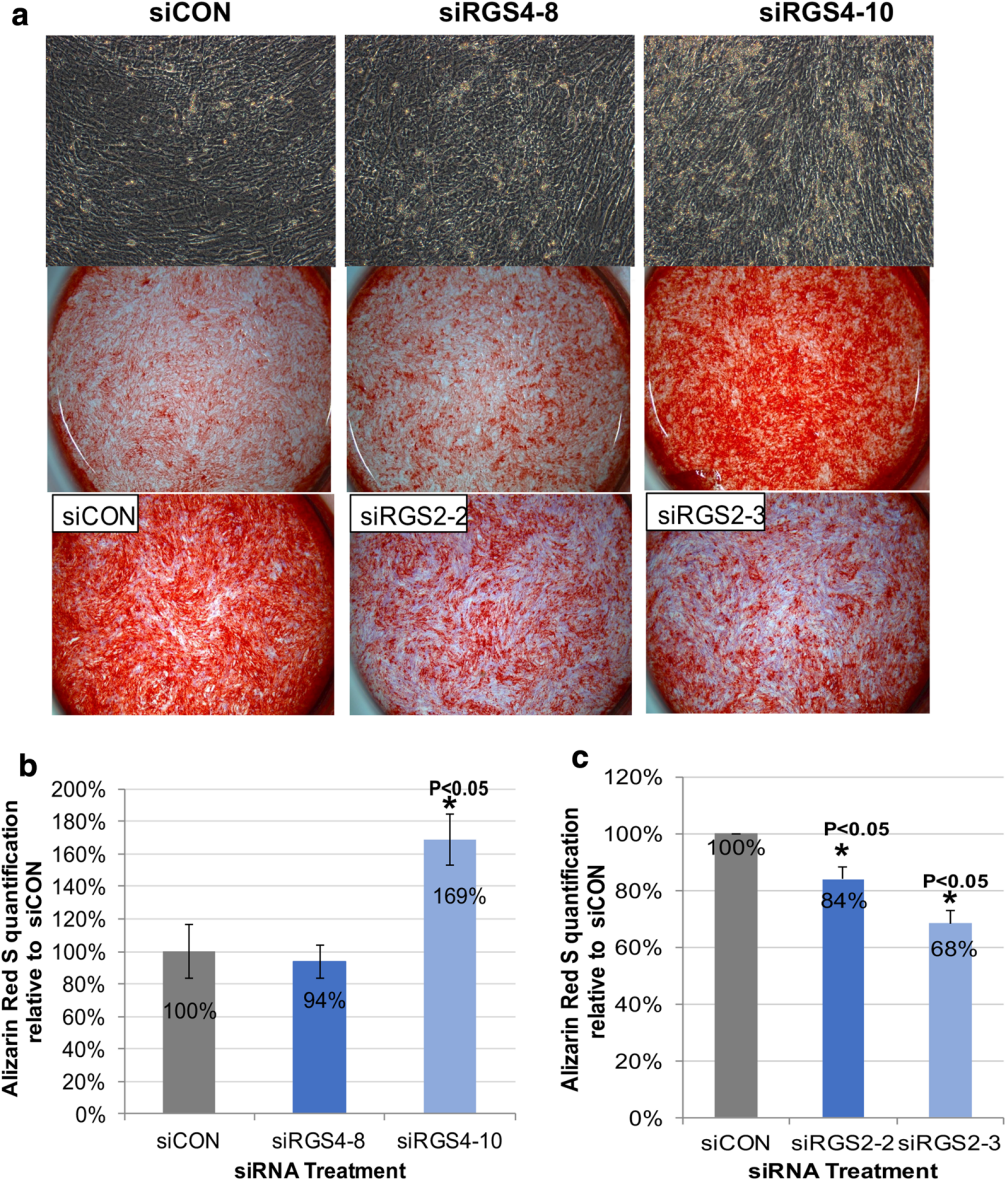
Effect of siRGS4 and siRGS2 on osteogenic differentiation of hMSCs. **a** Top row: Bright field images showing calcified deposits (yellowish color) in *siCON* and *siRGS4* treatment groups. Middle row: Bright field images showing alizarin red stained cells with calcified deposits in red color in *siCON* and *siRGS4* treatment groups. Bottom row: Bright field images showing alizarin red stained cells with calcified deposits in red color in *siCON* and *siRGS2* treatment groups. **b** Alizarin red S stain quantification in *siCON* and *siRGS4* treatment groups. **c** Alizarin red S stain quantification in *siCON* and *siRGS2* treatment groups. Imges and graphs represent the mean quantification of *siRGS* treatment wells set relative to that of *siCON* wells from a representative experimental set (n = 3). Comparison was made between *siCON* and *siRGS* treatment groups. *p < 0.05

In comparison to *siCON* treatments, there was increased as well as earlier onset of calcific deposit (day 11) present in *siRGS4*-*10* treatment groups but not so much in *siRGS4*-*8* groups (Fig. 9a, top row), which could also be visually confirmed by increased amount of Alizarin Red S stain in *siRGS4*-*10* treatment groups at the end of differentiation (day 18–24) (Fig. 9a, middle row). On the other hand, mineral deposit was decreased in both *siRGS2*-*2* and *siRGS2*-*3* samples compared to *siCON* groups (Fig. 9a, bottom row). Consistently, Alizarin Red S quantification was significantly higher in *siRGS4*-*10* treated samples (169%, p < 0.05) (Fig. 9b), but significantly lower in both *siRGS2*-*2* (84%, p < 0.05) and *siRGS2*-*3* (68%, p < 0.05) treated samples compared to *siCON* controls (100%) (Fig. 9c). Nuclear count revealed no significant difference between *siCON* and any *siRGS4* or *siRGS2* treatment groups (data not shown).

In conclusion, *siRGS4* and *siRGS2* had opposing effect on osteogenic differentiation of hMSCs, with the former promoting while the latter inhibiting the process, without affecting total cell numbers. In addition, similar to a more disruptive effect of *siRGS4*-*10* on adipogenic differentiation of hMSCs as compared to *siRGS4*-*8*, the former also demonstrated a greater enhancing effect on osteogenic differentiation of hMSCs compared to the latter.

### Effects of siRGS2 and siRGS4 on the expression of osteogenic markers

Since si*RGS4* promoted osteogenic differentiation of hMSCs while si*RGS2* inhibited it without affecting total cell numbers, it implied a direct effect on osteogenic differentiation. Effect of *siRGS4* and *siRGS2* on the temporal expression of known osteogenic markers, Runx2, Osteocalcin (OC) and alkaline phosphatase (ALP) was further evaluated. Runx2 is an osteogenic master regulator [14]. *OC* encodes a bone specific protein synthesized by osteoblast and serves as a marker of osteogenic maturation [59], while *ALP* encodes an enzyme that function to promote mineralization by increasing phosphate concentrations [60, 61]. Since type II/p57 isoform of Runx2 has been shown to be bone specific [62], primers amplifying specifically the N-terminal region of the gene that encodes the bone-specific MASNS polypeptide domain was used in analyzing the expression of Runx2.

**Fig. 10:**
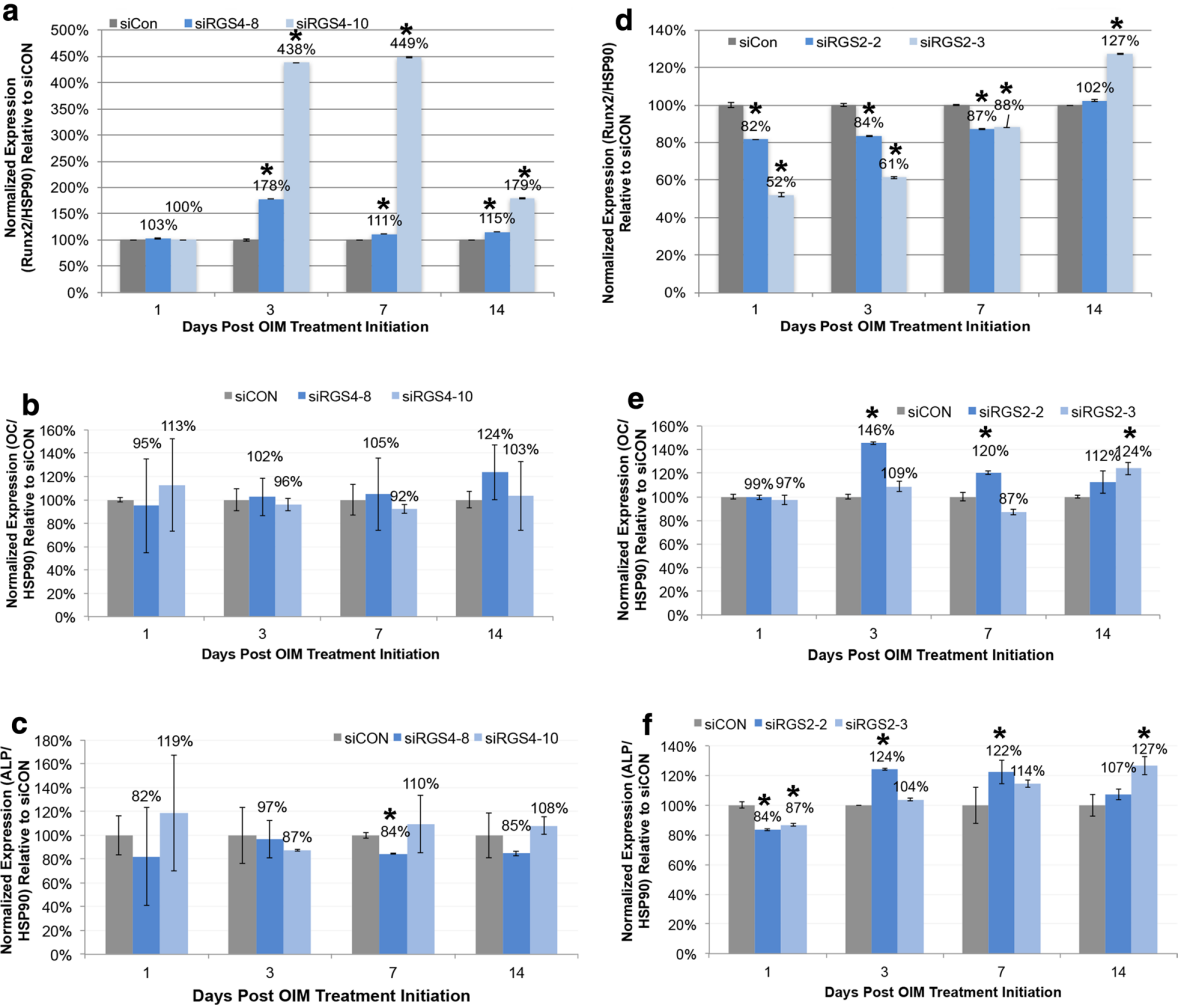
Effect of siRGS4 and siRGS2 on the expression of osteogenic markers. Expression of *Runx2*, *OC* and *ALP* were examined at day 1, 3, 7 and 14 post osteogenic treatment initiation in *siCON* and *siRGS4* (**a**–**c**) or *siRGS2* (**d**–**f**) transfected AD-hMSCs. Graphs represent average expression level of each gene normalized to that of *HSP90* and set relative to its normalized expression in *siCON* transfected cells. **a** Expression of *Runx2* in *siRGS4* and *siCON* transfected cells. **b** Expression of *OC* in *siRGS4* and *siCON* transfected cells. **c** Expression of *ALP* in *siRGS4* and *siCON* transfected cells. **d** Expression of *Runx2* in *siRGS2* and *siCON* transfected cells. **e** Expression of *OC* in *siRGS2* and *siCON* transfected cells. **f** Expression of *ALP* in *siRGS2* and *siCON* transfected cells. Error bars represent variation between independent repeats (n = 2). Expression comparison was made between *siCON* and *siRGS* treatment groups at each given time point. *p < 0.05

In *siRGS4* OIM treatments, *Runx2* expression was upregulated in both *siRGS4*-*8* and *siRGS4*-*10* treated cells as compared to *siCON* controls starting on D3 post OIM treatment initiation, but the enhancement is clearly much stronger in *siRGS4*-*10* samples, at about 2- to 4-fold higher level than in *siRGS4*-*8* (Fig. 10a), which is consistent with the greater effect of *siRGS4*-*10* on promoting osteogenic differentiation. For expression of OC, no significant difference between *siCON* and *siRGS4*-*8* or *siRGS4*-*10* samples was observed at any time point analyzed (Fig. 10b). Expression of *ALP* was also very similar between *siRGS4*-*8*/siRGS4-10 and *siCON* at all time points, except for day 7, when it was slightly down regulated in *siRGS4*-*8* (84%, p < 0.05) (Fig. 10c). Overall, *Runx2* was upregulated by both *siRGS4*-*8* and *siRGS4*-*10*, but at much greater level by the latter. *ALP* appeared to be transiently downregulated by *siRGS4*-*8* but remained unaffected by *siRGS4*-*10*.

In *siRGS2* OIM treatments, *Runx2* was down regulated by both *siRGS2*-*2* and *siRGS2*-*3* treatment starting at day 1 and lasted at least until day 7, at about 52–88% expression level of its normal expression (p < 0.05) in *siCON* (100%) (Fig. 10d). Expression of *OC* in *siRGS2*-*2* and *siRGS2*-*3* did not significantly differ from *siCON* treatment at day 1, but was transiently upregulated at day 3 (146%, p < 0.05) and day 5 (120%, p < 0.05) in *siRGS2*-*2* and at day 14 (124%, p < 0.05) in *siRGS2*-*3* compared to *siCON* (100%) (Fig. 10e). Finally, expression of *ALP* was slightly downregulated in *siRGS2*-*2* (84%) and *siRGS2*-*3* (87%) but then was transiently upregulated at day 3 (124%, p < 0.05) and day 5 (122%, p < 0.05) in *siRGS2*-*2* and at day 14 (127%, p < 0.05) in *siRGS2*-*3* compared to *siCON* (100%) (Fig. 10f). Overall, *Runx2* was slightly downregulated by both *siRGS2*-*2* and *siRGS2*-*3* from day 1 until at least day 7. *ALP* expression was also detected to be slightly downregulated on day 1, but was shifted to slight upregulation on later days. Expression of *OC* was also slightly upregulated by *siRGS2*-*2* or *siRGS2*-*3* at day 3 and 7 or day 14, respectively, following the same trend as *ALP* expression on those days.

In conclusion, *Runx2* was upregulated by both *siRGS4*-*8* and *siRGS4*-*10* throughout osteogenic differentiation but downregulated by *siRGS2*-*2* and *siRGS2*-*3*. *RGS4* silencing had no significant effect on the expression of *OC* or *ALP,* while *RGS2* silencing transiently downregulated *ALP* expression early on before upregulating it along with *OC* at later time points.

## Discussion

To the best of our knowledge, this is the first study analyzing the expression regulation and function of RGS proteins during human adipogenesis and osteogenesis by using hMSCs as an in vitro cellular model. RGS protein family contains over twenty members categorized into four subfamilies (R4/B, RZ/A, R7/C, and R12/D). Both RGS2 and RGS4 belong to the R4/B subfamily, along with RGS1, 3, 5, 8, 13, 16, 18 and 21 [63]. RGS proteins are intracellular proteins possessing GTPase activating protein (GAP) activity, which stimulates GTP hydrolysis of Gα subunit, leading to its re-association with the Gβγ of G proteins and termination of GPCR mediated signaling [37]. Both the Gα and the Gβγ dimer can go on to activate downstream effectors like adenylyl cyclase, phospholipase C (PLC-β), RhoA signaling, and ion channels [64, 65]. The duration of an activated GPCR-G protein is defined by the time that the Gα subunit is in its GTP-bound state [66], hence is regulated by the activities of RGS proteins. Gα subunits are subdivided into four subgroups based on sequence homology and effector selectivity: Gα_s_, Gα_i/o_, Gα_q_, Gα_12_ [67]. These Gα members can either activate or inactivate distinct downstream effectors and its selectivity of RGS proteins is dependent on sequence elements within and outside the RGS domain and the helical domain of Gα proteins [68, 69]. RGS2 possesses intrinsic GAP activity that is selective for G_q_-class Gα subunits, whereas RGS4 has intrinsic GAP activity for both G_q_ and G_i/o_-class Gα subunits [38, 39]. However, it is poorly understood as to how the specificity of RGS/GPCR coupling is achieved and how different RGS proteins might coordinate with each other in regulating the same biological events. Our study revealed an interestingly opposite gene expression pattern of RGS2 and RGS4 in response to adipogenic induction, which initially triggered our interest in further understanding their roles during human adipogenesis.

*RGS4* was expressed in high level in hMSCs but was quickly down regulated to near undetectable level within 24 h post adipogenic initiation. Expression knockdown of *RGS4* by *siRGS4* resulted in significantly reduced total cell numbers, indicating that it normally plays a role in regulating cell proliferation, and possibly a role in hMSCs self-renewal. Interestingly, down regulation of *RGS4* also inhibited adipogenic differentiation of hMSCs, indicating that it plays a positive role during adipogenesis, which seems to contradict with its down regulation in response to adipogenic induction. One could speculate that its down regulation during the first 3 days of adipogenic induction might be necessary for hMSCs to exit its ‘stem cell’ mode and prepare for differentiation, however, subsequent adipogenic commitment (day 3 and day 6 post adipogenic initiation) and maturation (after day 6) would benefit from up-regulation of *RGS4* expression. Molecular study indeed demonstrated that *siRGS4* inhibited the expression of *PPARγ* and *C/EBPα*, whose normal upregulation in response to adipogenic induction is concomitant with the onset of adipogenic commitment, suggesting that *RGS4* expression is beneficial to the upregulation of those two master control genes. It is also possible that the down regulation of *RGS4* during normal adipogenic differentiation of hMSCs might have exerted a negative effect throughout and maintaining its high level of expression as in undifferentiated hMSCs could have significantly facilitated the differentiation process. To distinguish the two different scenarios would require additional future study to effectively overexpress *RGS4* during adipogenic differentiation. Nevertheless, our observation of the positive role that RGS4 plays during adipogenesis is consistent with past study of *RGS4* knockout mice, which showed a significantly lower body weight compared to wild type mice [53], though in a separate study, the observed weight difference was contributed to increased catecholamine secretion in adrenal gland and consequently lipolysis in adipose tissue [54].

On the other hand, expression of RGS2 was very low in hMSCs but escalated to high level within 24 h of adipogenic initiation, which lasted until day 6 post adipogenic initiation before subsiding to the same expression level as in hMSCs. Our functional study of RGS2 also demonstrated that it normally played a positive role during adipogenic differentiation of hMSCs, as its expression knockdown led to decreased differentiation. However, unlike RGS4, knockdown of RGS2 did not appear to exert significant effect on the expression of known adipogenic marker genes including *PPARγ*, *C/EBPα* and *LPL*, suggesting that RGS2 might normally regulate adipogenesis through a different route. Nevertheless, the overall effect of *siRGS2* on adipogenesis is consistent with past findings from RGS2 knockout mice, which have lower weights, reduced fat deposits, decreased serum lipids, and lower leptin levels [50, 51]. It is interesting to note that there was greater phenotypic suppression on adipogenesis by *siRGS2* in HI FBS CM based AIM compared to Hyclone CM based AIM, which corresponded to a greater level of *RGS2* expression knockdown by *siRGS2* in the former vs. the latter condition. It is possible that expression knockdown is context dependent and may be more effective when the target gene’s overall expression level is lower.

While future studies are needed to understand the molecular mechanisms by which RGS2 and RGS4 might regulate adipogenesis, one could hypothesize a couple of potential mechanisms. Both G_q_- and G_i_-class Gα proteins can activate Rho (a subfamily of small GTPase proteins including RhoA) regulated signaling pathways involved in cytoskeletal remodeling, cell movement and organelle development [70–72]. RhoA-ROCK signaling plays important role in adipogenic commitment and ROCK inhibitor promotes adipogenesis [71]. Induction of adipogenic differentiation leads to disruption of actin stress fibres through downregulation of RhoA-ROCK signaling and increased monomeric G-actin and its association with MKL1, a transcriptional coactivator, which prevents the nuclear localization of MKL1 and allows subsequent expression of PPARγ [73]. It is plausible that attenuation of G_αi_ and/or G_αq_ activity by RGS4 or RGS2 is important for downregulating RhoA-ROCK signaling and allowing subsequent activation of PPARγ. Additionally, G_i_-class Gα proteins can inhibit adenylyl cyclase (AC) activity required for cAMP production. Increased cAMP level as the result of IBMX induction plays a key role in adipogenic commitment [17, 74]. By inactivating G_αi_, it is conceivable that RGS4 may allow the activation of AC and subsequent intracellular increase of cAMP to promote adipogenesis.

In addition, few past studies investigated the role of RGS proteins in osteogenesis. Bone remodeling involves bone reabsorption mediated by osteoclasts and bone production mediated by osteocytes. Several past studies have suggested a role of RGS proteins in bone remodeling. For example, RGS18 promotes osteoclastogenesis whereas RGS12 and RGS10 impairs it [48, 75, 76]. However no study thus far has examined the role of RGS in osteogenesis, though several GPCRs like parathyroid hormone 1 receptor (PTH1R), frizzled (Fz), and calcium sensing receptor (CaSR), which play important roles in osteoblast differentiation and function, are expressed in osteoblast and regulated by RGS proteins [49]. Activated PTH1R triggers the activation of G_αq_-PLC and G_αs_-AC signaling [77, 78]. G_q_-class Gα proteins can activate the phospholipase C-β (PLC-β) pathway that leads to the cleavage of phosphatidylinosiatol 4,5-biphosphate (PIP2) into inositol triphosphate (IP3) and diacylglycerol (DAG), and regulate intracellular Ca^2+^ release as well as protein kinase C (PKC) activity [77, 79]. CaSR acts as a calcium detector of extracellular Ca^2+^ and functions to maintain intracellular Ca^2+^ homeostasis through the G_αq/11_ pathway [80]. Frizzled receptors are activated by Wnt ligands, which triggers at least three distinct intracellular signaling cascades: beta-catenin pathway (canonical), Ca^2+^ pathway (noncanonical) and planar polarity pathway. All three pathways are implicated in bone formation [81, 82], with the canonical pathway leading to the expression of osteoblast-specific gene marker [83], Ca^2+^ pathway leading to intracellular Ca^2+^ increase [84], and the planar polarity pathway leading to the activation of Rho/Rac GTPases and cytoskeletal reorganization [85]. We demonstrated for the first time that RGS2 and RGS4 play apposing roles during osteogenic differentiation of hMSCs, with RGS4 as a negative regulator and RGS2 as a positive regulator. Their role was partly mediated by modulating the expression of known osteogenic regulator Runx2. However, it is also possible that the effect brought on by siRGS2 and siRGS4 was mediated through modulating the Gα protein activities downstream of the above mentioned GPCR mediated signals and potentially others as well to regulate the differentiation and maturation of osteoblasts. It is also interesting to note that the uncovered roles of RGS4 and RGS2 during osteogenesis concur with their expression pattern during normal osteogenic differentiation, with RGS4 downregulated and RGS2 upregulated upon osteogenic initiation. Our study demonstrates that RGS proteins are important regulators of bone remodeling by regulating not only osteoclastogenesis but also osteogenesis.

Lastly, it is interesting to point out that unlike RGS2, which demonstrated a clear correlation between its *mRNA* expression level and protein expression level in response to *siRGS2* transfection, down regulation of RGS4 appeared much delayed and weaker at the protein level as compared to the *mRNA* level in response to *siRGS4* transfection. This implies different expression regulation kinetics between RGS2 and RGS4, with the latter likely having long half-life that could mask the effect of total reduced *RGS4* transcripts. It is also possible that there might be unknown post-transcriptional regulation that hinders the progress of translation or post-translational protein modifications that might render the RGS4 antibodies used incapable of recognizing modified forms of RGS4, obscuring the actual total protein level. Poor correlation between expression levels of *mRNA* and protein level has been well documented [86–88], although in very few cases, the precise mechanisms have been investigated. This differential expression regulation of RGS2 and RGS4 adds another dynamic to the complexity of their roles during adipogenic and osteogenic differentiation of hMSCs.

GPCRs compose the largest family of membrane receptors and as a result, they are also the most widely targeted membrane proteins, with estimated 40% of clinical drugs targeting this system. As downstream regulators of these proteins, RGS proteins are likely to play essential roles during a wide range of developmental processes as well. During normal development, hMSCs residing in the adipose tissue as well as in the bone marrow would respond to different external stimuli by self-renewing or undergoing adipogenic or osteogenic differentiation. Some of these signals are mediated through the GPCR proteins. It is conceivable that RGS proteins might serve as factors of a feedback regulatory loop, in which that active differentiation would lead to expression change in these proteins such as RGS2 and RGS4, which in return would modulate the activity of their respective GPCR proteins in order to help attenuate/augment the cells’ further response to the external stimuli. How these proteins achieve regulatory specificity with different GPCRs and how they may interact with each other to fine tune specific biological event would be of great interest for future studies, as they are potentially druggable molecular targets for treating various physiological diseases.

## Conclusions

In summary, our results demonstrate that RGS2 and RGS4 are differentially regulated during adipogenic and osteogenic differentiation of hMSCs, with both playing positive roles during adipogenesis but opposing roles during osteogenesis. We demonstrated: (I) expression of RGS2 and RGS4 were found to be inversely regulated during adipogenesis, with RGS2 up-regulated and RGS4 down-regulated in response to adipogenic induction; (II) RGS2 expression was also up-regulated during osteogenesis, whereas RGS4 expression was down-regulated during the first 48 h of osteogenesis followed by up-regulation afterwards; (III) expression of RGS2 and RGS4 was regulated by DEX and IBMX independent of Insulin during adipogenesis, but only by DEX during osteogenesis; (IV) expression knock-down using si*RNA* against *RGS2* or *RGS4* both resulted in decreased adipogenic differentiation, though only knock-down of *RGS4* appeared to have significant effect on the expression of examined adipogenic markers including *C/EBPα* and LPL; and lastly, (V) expression knock-down of *RGS2* and *RGS4* resulted in decreased and increased osteogenic differentiation respectively, indicating that RGS2 is normally a positive regulator while RGS4 is a negative regulator during osteogenesis. Our study demonstrates for the first time that RGS2 and RGS4 are inversely regulated during human adipogenesis even though they both play positive roles, and on the other hand, both genes were also inversely regulated during early human osteogenesis (first 48 h) but play opposing roles. This implies that members of RGS proteins may play multifaceted roles during human adipogenesis and osteogenesis to balance or counterbalance each other’s function during those processes.

## Methods

### Cell culture

Human adipose-derived mesenchymal stem cells (ad-hMSCs; Fisher Scientific, cat# SV3010201) were cultured using Hyclone Advance STEM Mesenchymal Expansion Kit (Complete Media, Hyclone CM; Fisher Scientific, cat# SH30875KT) and grown and cultured in a 5% CO_2_ incubator at 37 °C. Cells were expanded at 1:5 splitting ratio using 0.05% trypsin–EDTA (Corning, cat# 25-02) and used at passage 4 for all assays.

### Clonogenicity assay

hMSCs at P4 passage were plated at 100 cells per 10-cm plate or 96 cells per 96-well plate (1 cell/well) and cultured continuously for 21 days, with media change every 3 days. Cells were then rinsed with PBS after medium removal and stained with 0.5% crystal violet (Sigma Aldrich, cat# 6158) dissolved in 20% methanol for 30 min at room Temperature (RT). Colonies containing in excess of 50 cells were counted using a Leica dissecting microscope.

### Immunostaining

hMSCs at P4 passage were fixed with 4% paraformaldehyde for 10 min, rinsed three times with PBS, permeated with 0.25% Triton X-100 in PBS for 10 min at RT, washed four times with PBS, 5 min each with gentle shaking, blocked with 1% BSA in PBS for 1 h at RT, incubated with primary antibodies diluted in blocking solution overnight at 4 °C, followed by incubation with secondary antibodies also diluted in blocking solution for 1 h at RT. Images were obtained using Olympus IX50 fluorescence microscope. Primary antibody against CD73 (dilution 1:5) and CD105 (dilution 1:12.5) were from Thermofisher (cat# 41-0200 and PA5-16895 respectively). Goat anti-mouse Alexa Fluor Plus 488 secondary antibody was from Thermofisher (cat# A32723) (dilution 1:500) and Donkey anti-Rabbit FITC secondary antibody (dilution 1:500) was from R&D (cat# 711-095-152).

### Flow cytometry

Detailed procedure can be found in our previously published study [89]. Briefly, hMSCs at P4 passage were collected and centrifuged at 1000 rpm for five minutes. The pellet was resuspended in 3 ml/well wash buffer (98% PBS + 2% Fetal Calf Serum) and counted with Countess Automated Cell Counter (C10227, Life Technologies). About 4.5 × 10^5^ cells in 100 μl were aliquoted into each FACS tube (coated with 1% BSA overnight at 4 °C prior), and 5 μl of each labeled primary antibody was added in each tube for staining for 30 min at 4 °C. Cells were stained with FITC anti-human CD90 (cat# 328107, Biolegend) alone, Pacific Blue anti-human CD73 (cat# 344011, Biolegend) alone, or both together for 30 min at 4 °C. FITC Mouse IgG1 (cat# 400109, Biolegend) was used as isotype controls. Unstained hMSCs were also used as negative controls. Cells were then fixed with 2% paraformaldehyde for 30 min at RT, washed with PBS once before flow cytometry analysis. Flow cytometry data was acquired through a Gallios flow cytometer (Beckman Coulter) at the City of Hope Analytical cytometry core and analyzed using the FlowJo software by Tree Star Inc.

### Cellular differentiation conditions

For all experimental assays, 64,000 cells were evenly plated in 24 well plates and media was changed every 48 h during differentiation induction unless specified otherwise. For adipogenic induction, ad-hMSCs were cultured in growth media supplemented with 0.2 or 1.0 μM dexamethasone (Dex; Sigma, cat# BCBP9963V), 0.45 μM 3-isobutyl-1-methylxanthine (IBMX; Sigma, cat# 15879), and 10 μg/ml insulin (Sigma Aldrich, cat# SLBN8658V) for 12–16 days. Depending on experimental needs, three different types of growth media were used: Hyclone CM, Heat-Inactivated Fetal Bovine Serum (FBS)-based complete media (HI-FBS CM), or FBS-based complete media (FBS CM). Hyclone CM refers to Hyclone Advance STEM Mesenchymal Expansion Kit used to grow and expand ad-hMSCs (Fisher Scientific, cat# SH30875KT), whose composition is undisclosed. HI-FBS CM and FBS CM were composed of High Glucose Dulbecco’s Modified Eagle’s Medium (DMEM), GlutaMAX (Gibco, cat# 10566-16), 1% non-essential amino acids (Gibco, cat# 11149-050), with 10% fetal bovine serum (FBS; Corning, cat# 35-010-CV) that was heat-inactivated or untreated, respectively. Heat-activation was performed in 57 °C water bath for 30 min. For osteogenic differentiation, ad-hMSCs were cultured in Hyclone CM supplemented with 0.2 or 1.0 μM dexamethasone, 10 mM β-glycerolphosphate (Sigma, cat# G9422), and 0.05 mM 2-phospho-l-ascorbic acid (Sigma, cat# BCBP8162V) for 18–26 days.

### SiRNA reverse transfections

Two transfection methodologies, referred to as reverse and forward transfection, were examined to optimize transfection efficiency. Forward transfection was achieved by equal plating of cells into culture vessel followed by introduction of *siRNA*-transfection agent complex at designed time point. Reverse transfection was achieved by introduction of *siRNA*-transfection reagent complex to culture vessel followed by equal plating of cells. Lyophilized *siRNA* at 1 nmol quantity was suspended in 100 µl of RNAase-free water obtaining a stock concentration of 10 µM, which was further diluted to 2 µM working stock (1 μl of 2 µM *siRNA* is equivalent to about 28 ng of *siRNA*). Transfection was done in 24-well plates. In reverse transfection, for each individual well, siRNA-transfection reagent complexes were prepared by adding 2.24 μl xtremeGENE *siRNA* transfection reagent (Roche, cat# 04476093001) into a tube containing 224 μl MEM basal media (Corning, cat# 10-022-CV), followed by the addition of 1.6 μl of 2 µM *siRNA* within 5 min. The complex was incubated for 25–30 min inside a cell culture hood at room temperature before transferring to a designated well. A total of 64,000 cells in 640 μl of growth media would be added to the mixture, followed by 24-h incubation in CO_2_ incubator before the media was exchanged for Hyclone CM growth media. The cells were further incubated for 24 h before subjecting to adipogenic or osteogenic differentiation induction. The following *siRNA* were used in this study: *siCON*: AllStars Neg. *siRNA* (Qiagen, cat# 1027284); *siRGS2*-*2*: Hs_RGS2_2 (Qiagen, cat# SI00045773); *siRGS2*-*3*: Hs_RGS2_3 (Qiagen, cat# SI00045780); *siRGS4*-*8*: Hs_RG4_8 (Qiagen, cat# SI03028018); and *siRGS4*-*10*: Hs_RG4_10 (Qiagen, cat# SI03097766).

### DAPI staining and total cell count

Nuclear staining was achieved using DAPI (4′,6-diamidino-2-phenylindole) nucleic acid stain (Sigma, cat# 108K4024). A working solution was prepared by diluting 1:3000 of a 14.3 mM DAPI stock in 1× PBS. Briefly, cells were fixed with 10% buffered formalin phosphate, washed three times with distilled water, and stained with the diluted DAPI solution for 30 min at room temperature. Cultures were then washed three times with distilled water and images were acquired using Olympus IX50 fluorescence microscope (7 images per well at 100× magnification were taken). Nuclear counts of each image were done using CellProfiler Image Analysis Software [90].

### Alizarin red staining and quantification

Mature osteocytes secrete calcium phosphate were detected 18–24 days after osteogenic induction using a 2% Alizarin Red S Staining solution (Acros Organic, cat# 130-22-3) (pH 4.1–4.3 adjusted with 0.5% Ammonium hydroxide). Cells were first fixed by 10% formalin-PBS, rinsed twice with water, incubated with Alizarin Red S solution for 10 min, washed four times with distilled water with 5 min intervals between wash, and air dried for later imaging and quantification. For quantification, a modified manufacturer’s protocol of an osteogenesis quantitation kit (Millipore, ECM815) was used. Briefly, dried stained cultures were incubated with 10% acetic acid for 20 min at room temperate. The loosely attached monolayer was then scrapped and total well content was transferred to microcentrifuge tubes. The mixtures were vortexed vigorously, parafilmed, and incubated at 85 °C for 10 min, followed by incubation on ice for 5 min and subsequent centrifugation at 20,000×*g* for 15 min. Supernatant was then transferred to a new microcentrifuge tube. Optical densities of solutions were measured at 405 and 690 nm in an ELx800 96 well plate reader (BioTek).

### Oil-Red-O staining and quantification

Lipid droplets in mature adipocytes can be identified using Oil-Red-O staining solution. After fixing with 10% buffered formalin phosphate (Fisher Scientific, cat# SF100-4), cells were incubated with 100% propylene glycol (Amresco, cat# 0575) for 5 min at room temperature, followed by its removal and staining with Whatman filter paper-filtered Oil Red O solution (Electron Microscopy Sciences, cat# 36609-01) for 30 min to 2 h with gentle rocking. Next, staining solution was removed and cells were incubated with 85% propylene glycol (Fisher Scientific, cat# A426P) for 5 min, followed by rinsing in distilled water three times. Stained cells would remain covered in distilled water, parafilmed and stored at 4 °C refrigerator. Whole-well images were taken using a Leica dissection microscope. For Oil-Red-O staining quantification, cells samples were air dried overnight after removing water and Oil-Red-O stain was extracted using 150 μl/well (24-well plate) 100% isopropyl alcohol for 1 min. Optical density readings for extracted stain solution were measured at 510 and 690 nm in an ELx800 96 well plate reader (BioTek).

### Adipocyte cell counts and area measurements of stained oil droplets

To determine total adipocyte and total cell counts, wells were imaged using an Olympus IX50 microscope at 100× magnification after double staining cultures with both DAPI and OilRedO stain. Images were taken starting from the bottom to the top of each individual well, resulting in seven separate fields of view spanning the entire well. DAPI stain showing nuclear stain were imaged using UV-light as excitation light (emission light: blue fluorescence) and OilRedO stain was imaged using green light as excitation light (emission light: red fluorescence). For adipocyte count, DAPI and OilRedO images taken in the same field of view were merged using Adobe Photoshop and mature adipocytes (identified by large concentration of oil droplets) were manually counted in Photoshop CS6 (Adobe).

For total area measurements of stained oil droplets, phase contrast images of OilRedO stained cells were taken and processed through an imaging analysis software (ImagePro Plus 7.2), which outlined the stained oil droplets of mature and immature adipocytes resulting in quantitative area measurements. Total area measurements of stained oil droplets were summed for each well and the average area measurement for each treatment group (12 wells/group) was then calculated and graphed relative to control group.

### RT-PCR analysis

For all cell pelleting, cells were detached with 0.05% Trypsin–EDTA, washed twice with 1× PBS and stored at – 80 °C until RNA extraction. Total RNA was isolated from all treatment groups using RNeasy Kit (Qiagen, cat# 74134). Equal concentrations of RNA (50 or 100 ng) was then reverse transcribed into cDNA using Superscript III Reverse Transcriptase Kit (Fisher Scientific, cat# 11752). PCR amplification was conducted using HotStarTaq Polymerase (Qiagen, cat# 203645). Primers for HPS90-beta (control), RGS2, RGS4, adipogenic markers (CEBPa, PPARy, & LPL), osteogenic markers (Osteocalcin, Runx2, and ALPL) and cell cycle markers (CDK1, CDK4, CCND1, CDK2) are listed in Table 1. PCR product gel images were obtained using SYBR safe DNA gel stain (Fisher Scientific, cat# 1760400) and Gel Doc XR+ system (BioRad). For all treatment groups, duplicates or triplicates as indicated were ran and analyzed. Quantifications are reported as average expression for each gene of interest normalized to HSP90 and made relative to control ± standard deviations.

**Table 1:** Primer sequences and associated PCR conditions

	Forward sequence	Reverse sequence	Product size (bps)	Annealing Tm (°C)
HSP90-beta	TACTTGGTGGCAGAGAAAGT	CTCATCTGAACCCACATCTT	441	60
RGS2	CAAACAGCCGGGACTCCAGC	TGCTGGCATGCAGCTGGTCA	303	63.5
RGS4	TCCCTGGTCCCTCAGTGTGCC	AAGCATGCCCTGAGCACCCA	958	64
PPARγ	AAGCCCTTCACTACTGTTGA	ACCTGATGGCATTATGAG AC	444	56
CEBPα	CCTAAGGTTGTTCCCCTAGT	GAGAGTCTCATTTTGGCAAG	547	58
LPL	GTCCGTGGCTACCTGTCATT	AGCCCTTTCTCAAAGGCTTC	717	60
Runx2	TCTTCACAAATCCTCCCC	TGGATTAAAAGGACTTGGTG	230	55
OC	CTACCTGTATCAATGGCTG	CAGATTCCTCTTCTGGAGTTTA	310	56
ALPL	TGGAGCTTCAGAAGCTCAACA	ATCTCGTTGTCTGAGTACCAG	450	60
CDK1	GGATCTACCATACCCATTGAC	CCATGTACTGACCAGGAGGG	327	55
CDK2	TGACTCGCCGGGCCCTATTCC	CCCAAGGCCAAGCCTGGTCA	381	60
CDK4	AGTTTCCGCGCGCCTCTTTG	GGCACAGACGTCCATCAGCCG	455	63
CCND1	CTCCAGAGGGCTGTCGG	CTCGGCCGTCAGGGGGA	528	60
Runx2/p57	CGC CTC ACA AAC AAC CAC AG	TCA CTG TGC TGA AGA GGC TG	225	60

### Western Blot

Protein was extracted from whole cell lysis from 5.6 × 10^5^ cells per treatment group on day 1, 2, 3, 4, 5 or 7 (for RGS4 detection), or day 1, 2 or 3 (for RGS2 detection) post AIM or OIM treatment initiation. Briefly, Protein was extracted using M-PER reagent (Thermofisher cat# 78501) containing 1× Halt protease inhibitor (Thermofisher cat# 87785). About 20 μg of protein per sample was loaded and separated in NuPAGE 10% bis–tris gel (Thermofisher cat# NP0301BOX). After transferring to nitrocellulose membrane, the blot was incubated in antigen pretreatment solution (SuperSignal Western Blot Enhancer kit, Thermofisher cat# 46640) for 10 min before incubating in StartingBlock blocking buffer (Thermofisher cat# 37543) for 30 min at RT, followed by incubation with primary antibody raised in mouse (Santa Cruz Biotechnology, HSP90 F-8, sc-13119; RGS4 H-12, sc398348; RGS4 D-8, sc398658; and RGS2 BC-43, sc-100761) and HRP-conjugated anti-mouse secondary antibody (Thermofisher, Cat# 31503) for 1 h each at RT with gentle shaking. RGS4 H-12 detects isoform 3 (34 kDa) whereas RGS D-8 detects isoforms 1 and 2 (23 kDa). To detect protein bands, blot was incubated in chemiluminescent substrate working solution (SuperSignal West Pico PLUS Chemiluminescent substrate kit, Thermofisher cat# 34577) overnight at 4 °C, and images were acquired and bands were quantified using Gel Doc XR+ system (BioRad).

### Statistical analysis

All graph data is presented as the average ± standard deviations (SD). Differences in data was considered significant if p < 0.05 as determined by student’s unpaired t test. p value was determined by comparing original raw data sets between treatment and control groups, prior to percentage calculation set relative to *siCON* as shown in graphs.

## Additional files

**Additional file 1: Figure S1.** Clonogenic Assay of Adipose-Derived hMSCs. The number of clones with more than 50 cells after 21 days of culture was counted and representative images of clones and non-clones are included as well. Error bars represent variation among triplicates in each independent repeat (n = 3).

**Additional file 2: Figure S2.** Expression Profile of hMSC markers CD73, CD90 and CD105 by Immunostaining and Flow Cytometry. Expression of both CD73 and CD105 were detected in ≥ 95% of the cells. Expression of CD73 was further confirmed by flow cytometry and co-staining with CD90, which also showed expression in ≥ 95% of the cells.

**Additional file 3: Figure S3.** Temporal Expression of RGS4 and RGS2 in HI-FBS CM based AIM and OIM Treatments. Expression of RGS2 and RGS4 was examined by RT-PCR in hMSCs cultured in 8 different media treatments, including Hyclone CM, HI-FBS CM, Hyclone CM based DEX media (Hyclone 1.0 µM DEX), HI-FBS CM based DEX media (HI-FBS 1.0 µM DEX), Hyclone CM based AIM media with 1.0 µM DEX (Hyclone 1.0 µM DEX AIM), FBS CM based AIM media with 1 µM DEX (FBS 1.0 µM DEX AIM), HI-FBS CM based AIM media with 1.0 µM DEX (HI-FBS 1.0 µM DEX AIM), and Hyclone CM based OIM media with 1.0 µM DEX (Hyclone 1.0 µM DEX OIM). Expression in each treatment condition was examined at six different time points, including D0.5, D1, D1.5, D2, D3 and D4 post initial treatment. **A** Graph of *RGS4* expression. **B** Graph of *RGS2* expression. Graphs represent average gene expression level normalized to that of *HSP90* and set relative to CM control at each given time point (n = 2).

**Additional file 4: Figure S4.** Efficiency of siRNA transfection in adipose-derived hMSCs. Ad-hMSCs were reverse transfected with either a scrambled *siCON* or *siTOX* at 16.5 nM. **A** Bright field images of transfected cells at day 1, 2, 6 and 12 post *siRNA* transfection. **B** Total live cells were determined using an automated cell counter at day 1, 2, 6 or 12 days post *siRNA* transfection. Graphed data is shown as mean ± SD (n = 3). Asterisks represent significant differences between *siTOX* and *siCON* treated cells (**p < 0.01).

**Additional file 5: Figure S5.** Expression knockdown of RGS4 and RGS2 at the protein level by siRGS4 and siRGS2, respectively. **A**. Western blot demonstrating expression of RGS4 detected by two different antibodies that recognized isoform 3 (34 kDa) and isoforms 1 & 2 (23 kDa) respectively in both *siRGS4* and *siCON* treatment groups on day 7 post OIM initiation. (n = 2). **B** Western blot demonstrating expression of RGS2 detected by its antibody that recognized all isoforms at around 26 kDa in both *siRGS2* and *siCON* treatment groups on day 2 post OIM initiation (n = 2).

**Additional file 6: Figure S6.** Expression knockdown of RGS2 mRNA by siRGS2 during adipogenic differentiation of hMSCs induced by HI-FBS CM based adipogenic media. Expression of RGS2 was examined at day 1, 3, 5, 7, and 14 after differentiation initiation at 48 h post siRGS2 transfection. **A** Expression level of RGS2 in each treatment group was determined relative to their expression in siCON control group, after normalization against internal control HSP90 at each given time point. **B** Agarose gel images of RGS2 and HSP90 RT-PCR products were shown. Error bars represent variation between independent repeats (n = 2). *p < 0.05, **p < 0.01. Expression comparison was made between siCON and siRGS2 treatment groups at each time point.
