# Supplementary figures and images for: Expression regulation and functional analysis of RGS2 and RGS4 in adipogenic and osteogenic differentiation of human mesenchymal stem cells
Source: Biol Res. 2017 Dec 26;50:43. doi: 10.1186/s40659-017-0148-1 (PMC5742872; doi:10.1186/s40659-017-0148-1)

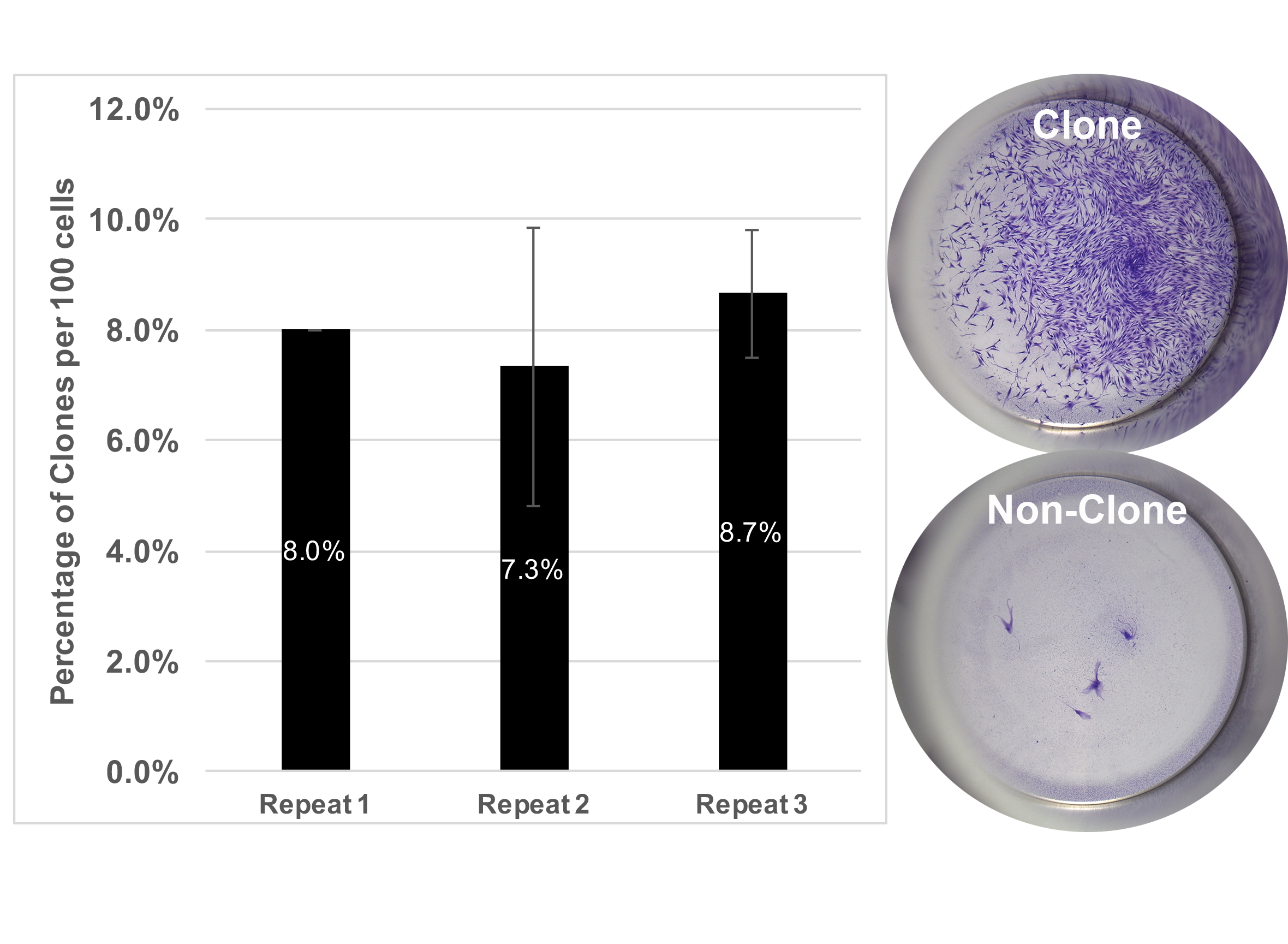

Supplement: Supplementary file 1 — Additional file 1: Figure S1. Clonogenic Assay of Adipose-Derived hMSCs. The number of clones with more than 50 cells after 21 days of culture was counted and representative images of clones and non-clones are included as well. Error bars represent variation among triplicates in each independent repeat (n = 3). [file 40659_2017_148_MOESM1_ESM.tif]

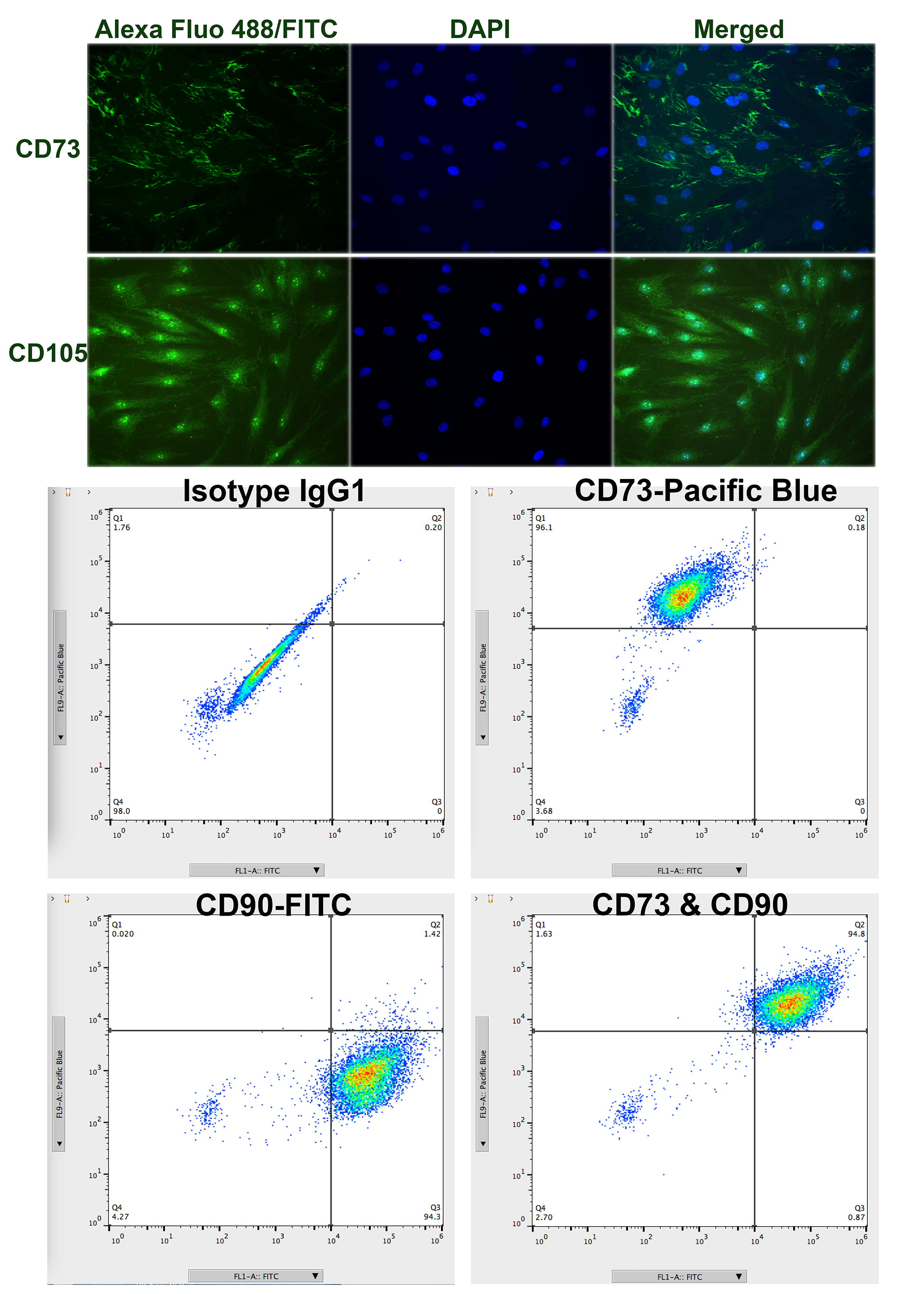

Supplement: Supplementary file 2 — Additional file 2: Figure S2. Expression Profile of hMSC markers CD73, CD90 and CD105 by Immunostaining and Flow Cytometry. Expression of both CD73 and CD105 were detected in ≥ 95% of the cells. Expression of CD73 was further confirmed by flow cytometry and co-staining with CD90, which also showed expression in ≥ 95% of the cells. [file 40659_2017_148_MOESM2_ESM.tif]

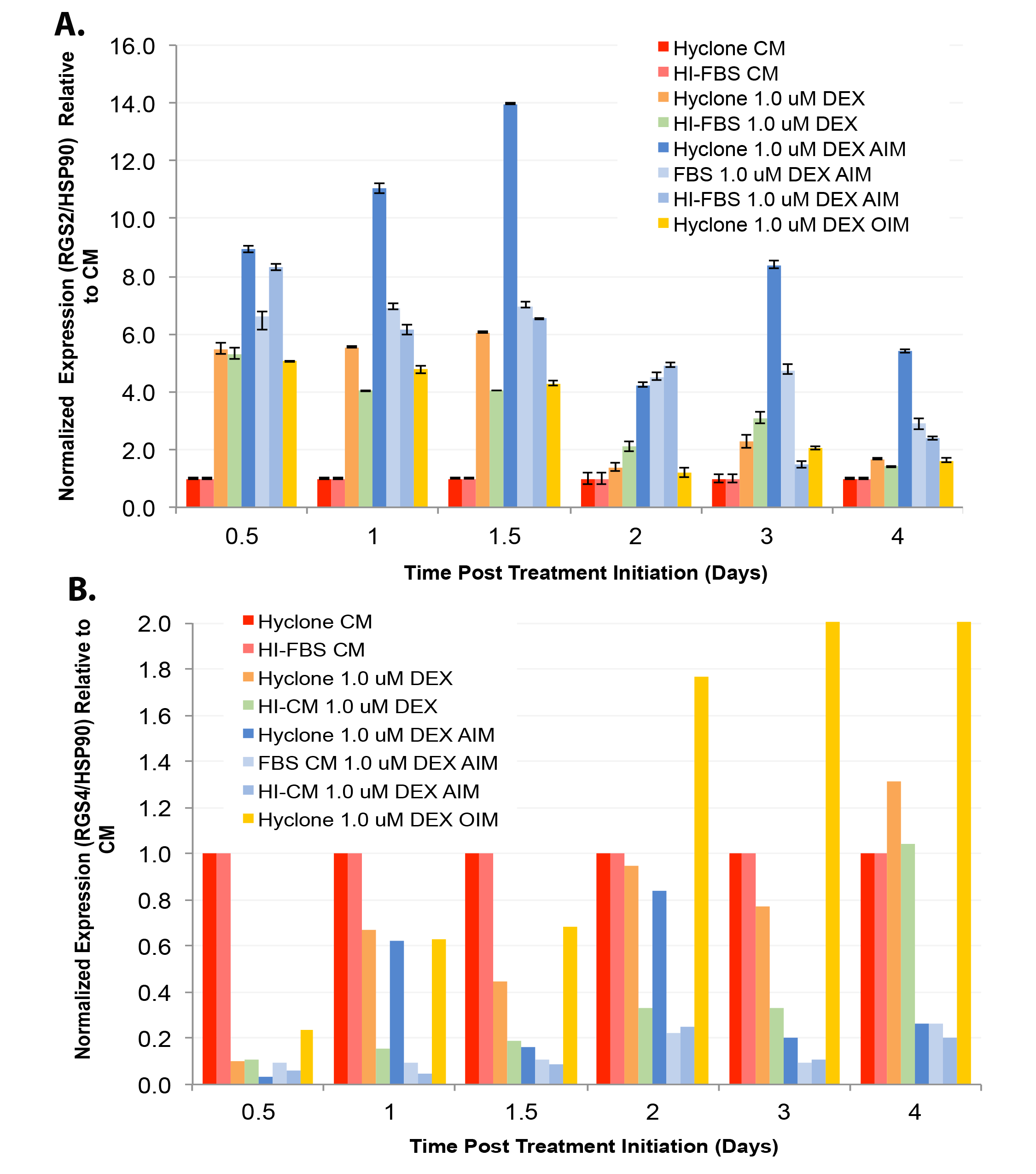

Supplement: Supplementary file 3 — Additional file 3: Figure S3. Temporal Expression of RGS4 and RGS2 in HI-FBS CM based AIM and OIM Treatments. Expression of RGS2 and RGS4 was examined by RT-PCR in hMSCs cultured in 8 different media treatments, including Hyclone CM, HI-FBS CM, Hyclone CM based DEX media (Hyclone 1.0 µM DEX), HI-FBS CM based DEX media (HI-FBS 1.0 µM DEX), Hyclone CM based AIM media with 1.0 µM DEX (Hyclone 1.0 µM DEX AIM), FBS CM based AIM media with 1 µM DEX (FBS 1.0 µM DEX AIM), HI-FBS CM based AIM media with 1.0 µM DEX (HI-FBS 1.0 µM DEX AIM), and Hyclone CM based OIM media with 1.0 µM DEX (Hyclone 1.0 µM DEX OIM). Expression in each treatment condition was examined at six different time points, including D0.5, D1, D1.5, D2, D3 and D4 post initial treatment. A Graph of RGS4 expression. B Graph of RGS2 expression. Graphs represent average gene expression level normalized to that of HSP90 and set relative to CM control at each given time point (n = 2). [file 40659_2017_148_MOESM3_ESM.tif]

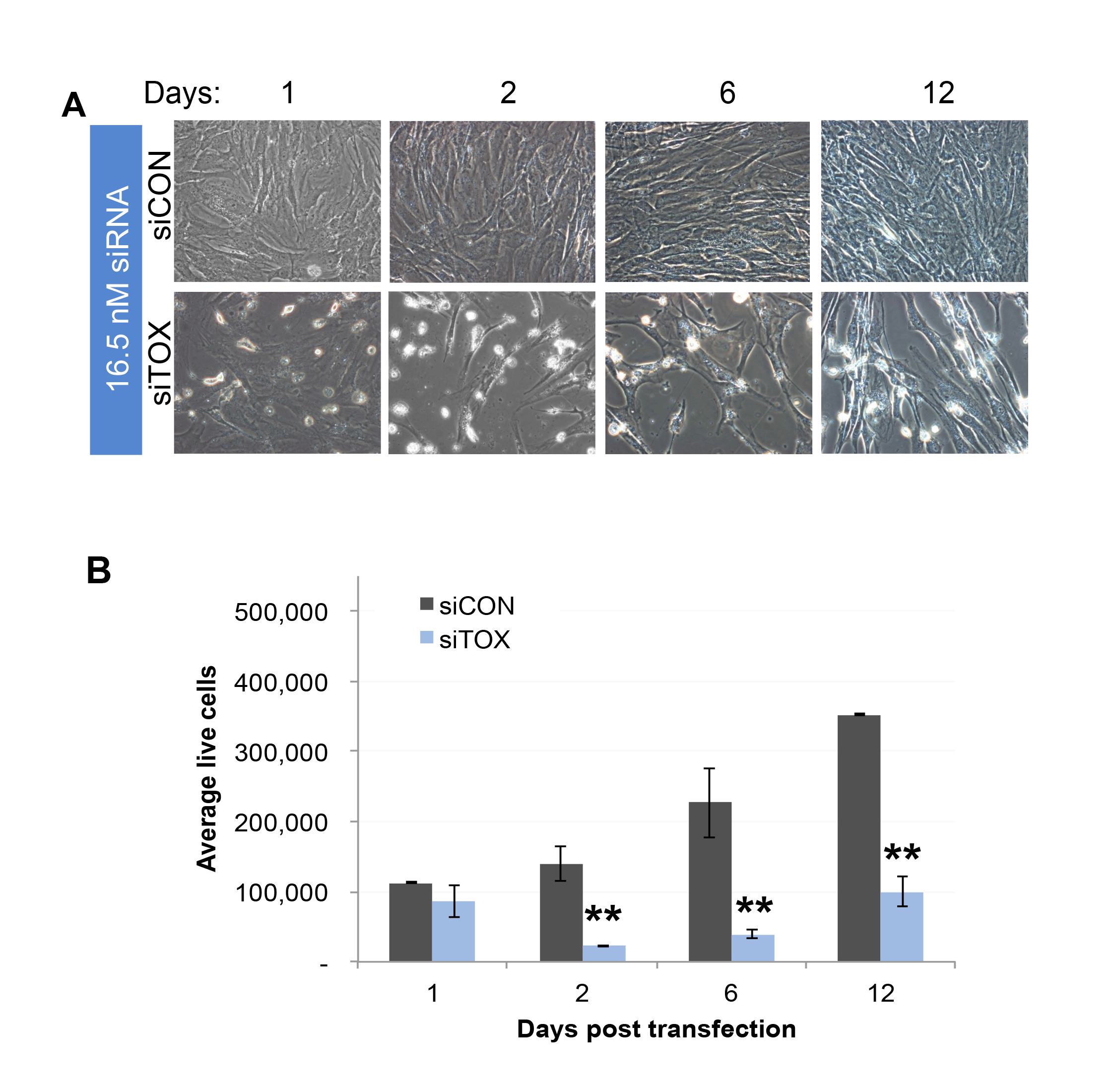

Supplement: Supplementary file 4 — Additional file 4: Figure S4. Efficiency of siRNA transfection in adipose-derived hMSCs. Ad-hMSCs were reverse transfected with either a scrambled siCON or siTOX at 16.5 nM. A Bright field images of transfected cells at day 1, 2, 6 and 12 post siRNA transfection. B Total live cells were determined using an automated cell counter at day 1, 2, 6 or 12 days post siRNA transfection. Graphed data is shown as mean ± SD (n = 3). Asterisks represent significant differences between siTOX and siCON treated cells (**p < 0.01). [file 40659_2017_148_MOESM4_ESM.tif]

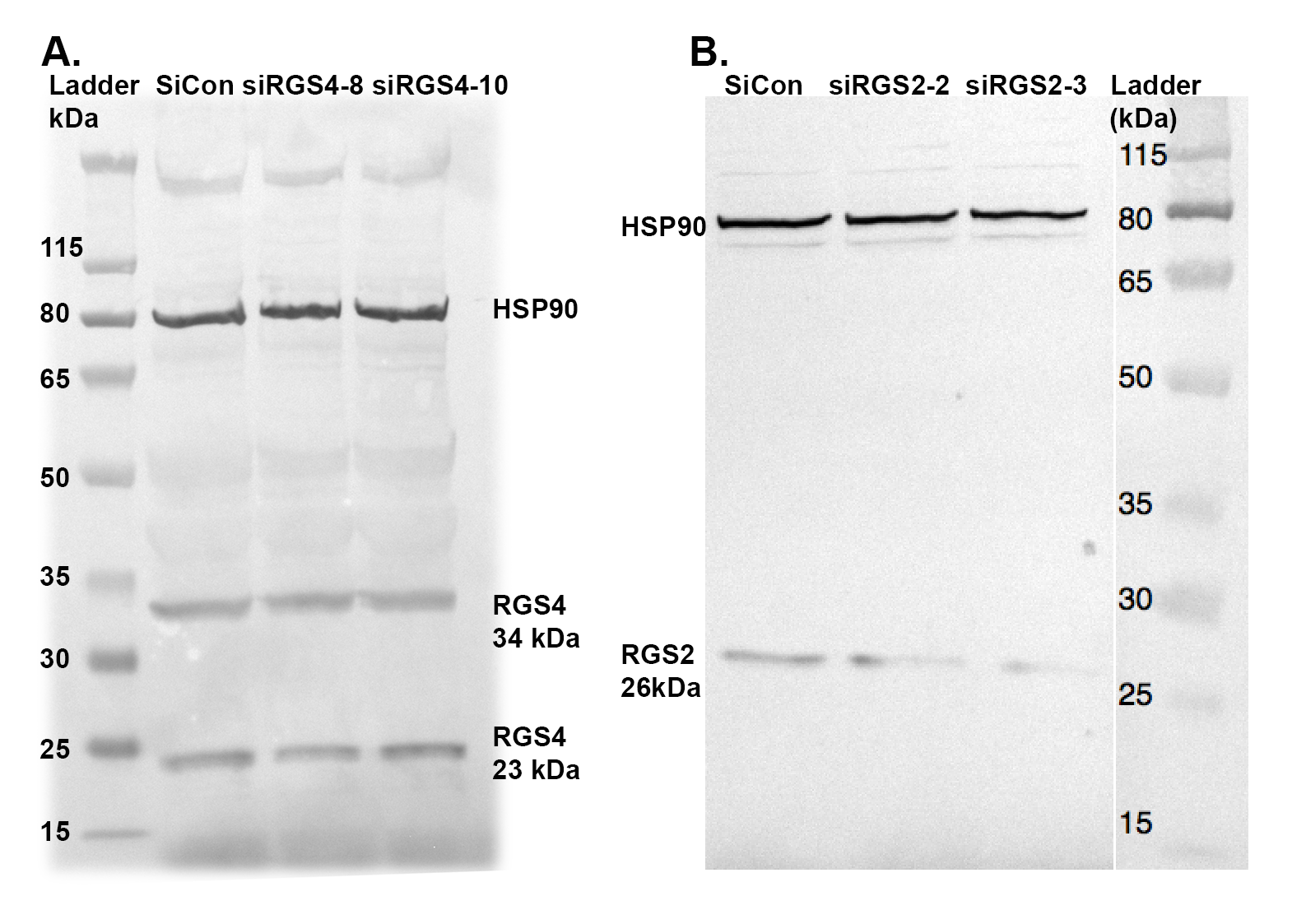

Supplement: Supplementary file 5 — Additional file 5: Figure S5. Expression knockdown of RGS4 and RGS2 at the protein level by siRGS4 and siRGS2, respectively. A. Western blot demonstrating expression of RGS4 detected by two different antibodies that recognized isoform 3 (34 kDa) and isoforms 1 & 2 (23 kDa) respectively in both siRGS4 and siCON treatment groups on day 7 post OIM initiation. (n = 2). B Western blot demonstrating expression of RGS2 detected by its antibody that recognized all isoforms at around 26 kDa in both siRGS2 and siCON treatment groups on day 2 post OIM initiation (n = 2). [file 40659_2017_148_MOESM5_ESM.tif]

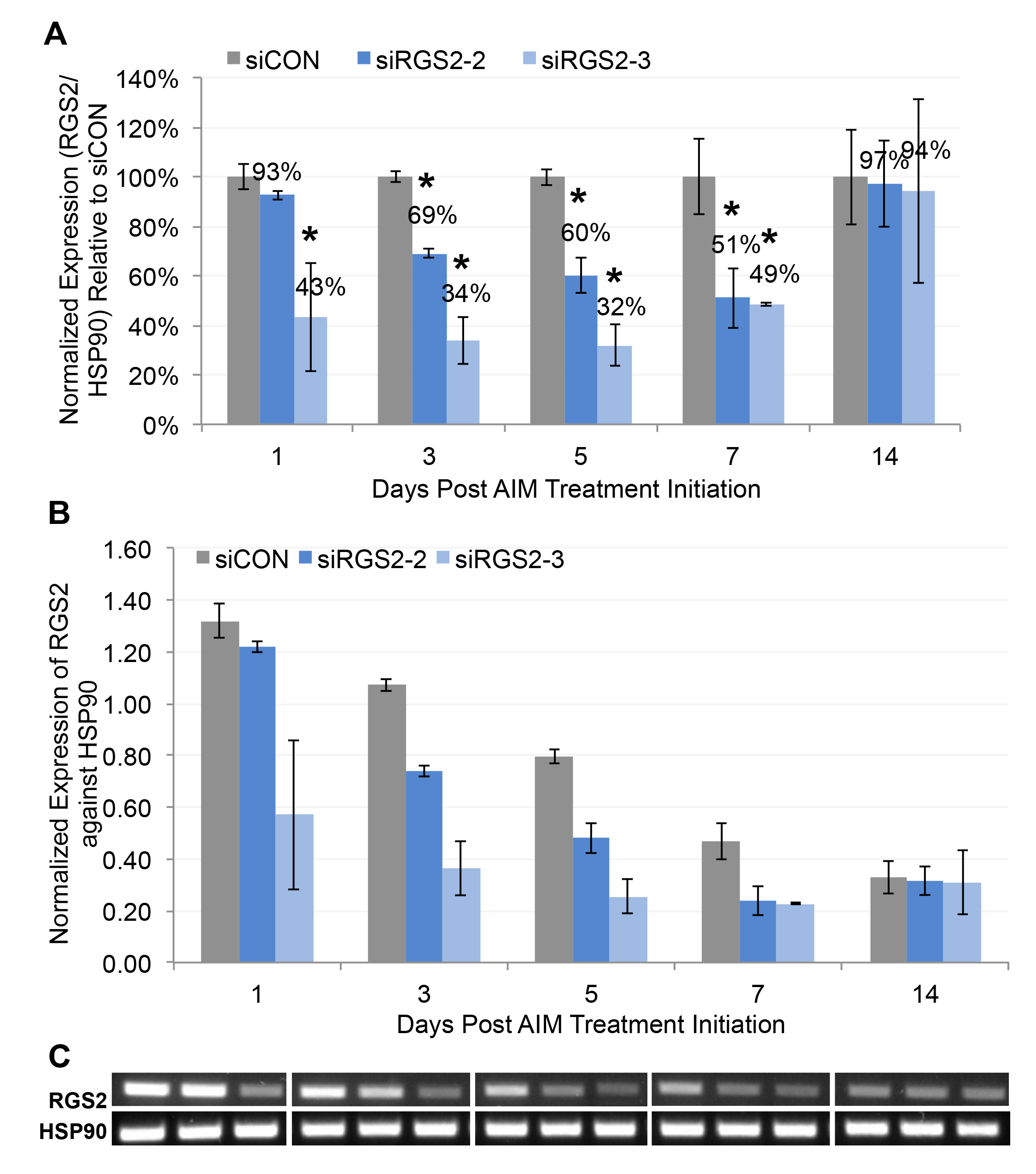

Supplement: Supplementary file 6 — Additional file 6: Figure S6. Expression knockdown of RGS2 mRNA by siRGS2 during adipogenic differentiation of hMSCs induced by HI-FBS CM based adipogenic media. Expression of RGS2 was examined at day 1, 3, 5, 7, and 14 after differentiation initiation at 48 h post siRGS2 transfection. A Expression level of RGS2 in each treatment group was determined relative to their expression in siCON control group, after normalization against internal control HSP90 at each given time point. B Agarose gel images of RGS2 and HSP90 RT-PCR products were shown. Error bars represent variation between independent repeats (n = 2). *p < 0.05, **p < 0.01. Expression comparison was made between siCON and siRGS2 treatment groups at each time point. [file 40659_2017_148_MOESM6_ESM.tif]
